# Interpretability and fairness evaluation of deep learning models on MIMIC-IV dataset

**DOI:** 10.1038/s41598-022-11012-2

**Published:** 2022-05-03

**Authors:** Chuizheng Meng, Loc Trinh, Nan Xu, James Enouen, Yan Liu

**Affiliations:** Department of Computer Science, University of Southern California, Los Angeles, CA 90089 USA

**Keywords:** Medical research, Outcomes research, Medical ethics, Computer science

## Abstract

The recent release of large-scale healthcare datasets has greatly propelled the research of data-driven deep learning models for healthcare applications. However, due to the nature of such deep black-boxed models, concerns about interpretability, fairness, and biases in healthcare scenarios where human lives are at stake call for a careful and thorough examination of both datasets and models. In this work, we focus on MIMIC-IV (Medical Information Mart for Intensive Care, version IV), the largest publicly available healthcare dataset, and conduct comprehensive analyses of interpretability as well as dataset representation bias and prediction fairness of deep learning models for in-hospital mortality prediction. First, we analyze the interpretability of deep learning mortality prediction models and observe that (1) the best-performing interpretability method successfully identifies critical features for mortality prediction on various prediction models as well as recognizing new important features that domain knowledge does not consider; (2) prediction models rely on demographic features, raising concerns in fairness. Therefore, we then evaluate the fairness of models and do observe the unfairness: (1) there exists disparate treatment in prescribing mechanical ventilation among patient groups across ethnicity, gender and age; (2) models often rely on racial attributes unequally across subgroups to generate their predictions. We further draw concrete connections between interpretability methods and fairness metrics by showing how feature importance from interpretability methods can be beneficial in quantifying potential disparities in mortality predictors. Our analysis demonstrates that the prediction performance is not the only factor to consider when evaluating models for healthcare applications, since high prediction performance might be the result of unfair utilization of demographic features. Our findings suggest that future research in AI models for healthcare applications can benefit from utilizing the analysis workflow of interpretability and fairness as well as verifying if models achieve superior performance at the cost of introducing bias.

## Introduction

With the release of large scale healthcare datasets, research of data-driven deep learning methods for healthcare applications demonstrates their superior performance over traditional methods on various tasks, including mortality prediction, length-of-stay prediction, phenotyping classification and intervention prediction^1–3^. However, deep learning models have been treated as black-box universal function approximators, where prediction explanations are no longer available as their traditional counterparts, e.g., Logistic Regression and Random Forests. Lack of interpretability hinders the wide application of deep learning models in critical domains like healthcare. In addition, due to bias in datasets or models, decisions made by machine learning algorithms are prone to be unfair, where an individual or a group is favored compared with the others owing to their inherent traits. As a result, more and more concerns about interpretability, fairness and biases have been raised recently in the healthcare domain where human lives are at stake^4^. These concerns call for careful and thorough analyses of both datasets and algorithms. In this work, we focus on the latest version (version IV^5^) of a widely used large scale healthcare dataset MIMIC^6^, and conduct comprehensive analyses of model interpretability, dataset bias, algorithmic fairness, and the interaction between interpretability and fairness.

*Interpretability evaluation* First, we benchmark the performance of common interpretability methods for feature importance estimation on multiple deep learning models trained for the mortality prediction task. Due to the complexity of dynamics in electronic health record data, there is no access to the ground truth of feature importance. Therefore, we utilize ROAR (remove and retrain)^7^ to quantitatively evaluate different feature importance estimations. On all models considered, the ArchDetect^8^ outperforms other interpretation methods in feature importance estimation. Then we qualitatively analyze the feature importance estimation results given by ArchDetect, and verify its effectiveness based on the observations that it distinguishes crucial features for mortality prediction. Importantly, we also observe that: (1) ArchDetect can recognize critical features not appearing in domain knowledge for mortality prediction. (2) Demographic features are important for prediction, which leads to our following audits of dataset bias and algorithmic fairness.

*Dataset bias and algorithmic fairness* We adopt the following commonly used demographic features as protected attributes: (1) *ethnicity*, (2) *gender*, (3) *marital status*, (4) *age*, and (5) *insurance type*. For dataset bias, we analyze the average adoption and duration of five types of ventilation treatment on patients from different groups. There exists treatment disparity among patient groups split by different protected attributes. However, multiple confounders may lead to the observed disparity in treatment. For algorithmic fairness, we evaluate the performance of state-of-the-art machine learning approaches for mortality prediction in terms of AUC-based fairness metrics. Experiment results indicate a strong correlation between mortality rates and fairness: machine learning approaches tend to obtain lower AUC scores on groups with higher mortality rates. Moreover, we find that prediction models trained with the MIMIC-IV dataset rely on racial attributes unequally across subgroups.

*Interactions between interpretability and fairness* We examine the interaction of interpretability and fairness by drawing connections between feature importance and fairness metrics, which is an understudied area in the community. We observe substantial disparities in the importance of each demographic feature used for in-mortality prediction across the protected subgroups, which raises a concern about how these demographic features should be used fairly in mortality prediction.

In summary, our main contributions are:

1. We have conducted a comprehensive analysis on a diverse set of popular interpretability methods for deep neural networks in the healthcare setting. We specifically focuse on the in-hospital mortality prediction task where interpretability is a must and evaluate all models and interpretability methods on the recently released large-scale MIMIC-IV dataset.
2. On interpretability, we find that the feature importance estimation results successfully identify most critical features in domain knowledge and recognizes new ones. We also find that deep methods rely on some demographic features for prediction. On fairness, our findings show that there exists treatment disparity among patient groups, and that in-hospital mortality predictors trained with MIMIC-IV can rely on racial attributes unequally across subgroups. In the end, we connect interpretability and fairness to show that feature importance from interpretability methods can help to identify potential biases in deep predictive models.
3. Our findings suggest that future research in AI models for healthcare applications can avoid the lopsided focus on prediction performance via analyzing the interpretability and fairness of models, as well as verifying if models reach good performance while introducing bias.

## Related work

### Interpretability evaluation

#### Interpretability of deep learning models

Due to the complexity of deep learning models, interpretability research has developed diversely, and many methods have been used to interpret how a deep learning model works from various aspects, including: (1) *Feature importance estimation*^9–19^. For a given data sample, these methods estimate the importance of each input feature with respect to a specified output. (2) *Feature interaction attribution*^8,20–24^. In addition to estimating the importance of individual features, these methods analyze how interactions of feature pairs/groups contribute to predictions. (3) *Neuron/layer attribution*^19,25–28^. These methods estimate the contribution of specified layers/neurons in the model. (4) *Explanation with high-level concepts*^29–31^. These methods interprete deep learning models with human-friendly concepts instead of the importance of low-level input features. In this paper, we focus on feature importance estimation due to its importance and the completeness of its evaluation methods.

#### Evaluation of feature importance interpretation

Since feature importance estimation assigns an importance score for each input feature, the evaluation of results is equivalent to the evaluation of binary classification results when the ground truth of feature importance is available, where the label indicates whether the feature is important for the problem^32^. constructs synthetic datasets with feature importance labels for evaluation^33^. obtains feature importance labels from both manually constructed tasks and domain experts^34^. derives importance labels from tasks with graph-valued data with computable ground truths. However, these evaluation methods require the accessibility of ground truth labels, which is hard to fulfill and is usually the problem itself we need to solve in domains such as healthcare.

For evaluation without ground truth, A common strategy to evaluate feature importance estimation is to measure the degradation of model performance with the gradual removal of features estimated to be important^35^. Perturbates features ranked by importance in test samples and calculates the area over the MoRF curve (AOPC): a higher AOPC means the information disappears faster with feature removal and indicates a better importance estimation^7^. Remove features from the entire dataset and retrain the model when obtaining AOPC, which excludes the interference of data distribution shifting^32^. Replace features with known feature distributions for evaluation on synthetic tasks to ensure the consistency of data distribution. In this paper, we utilize the evaluation in^7^.

### Fairness evaluation

#### Bias and fairness in machine learning

With the open access to large-scale datasets and the development of machine learning algorithms, more decisions in the real world are made by machine learning algorithms with or without human’s intervention, e.g., job advertisements promoting^36^, facial recognition^37^, treatment recommendation^38^, etc. Due to bias in datasets or models, decisions made by machine learning algorithms are prone to be unfair, where an individual or a group is favored compared with the others owing to their inherent traits. One well-known example is the software COMPAS (Correctional Offender Management Profiling for Alternative Sanctions), which was found a bias against African-Americans to assign a higher risk score of recommitting another crime than to Caucasians with the same profile^39^.

Based on the general assumption that the algorithm itself is not coded to be biased, the decision unfairness can be attributed to biases in the data, which is likely to be picked up and amplified by the trained algorithm^40^. Three major sources of data biases are^40^: (1) *Biased Labels:* the ground-truth labels for the machine learning algorithms to predict are biased; (2) *Imbalanced representation:* imbalanced representation of different demographic groups occurs when some protected groups are underrepresented with fewer observations in the dataset compared with other groups; (3) *Data Quality Disparity:* data from protected groups might be less complete or accurate during data collecting and processing. Mostly widely considered traits, such as gender, age, ethnicity, marital status, are considered as protected or sensitive attributes in literature^41^. Fairness has been defined in various ways considering different contexts or applications, two of them are the most widely leveraged for bias detection and correction: *Equal Opportunity*, where the predictions are required to have equal true positive rate across two demographics, and *Equalized Odds*, where an additional constraint is put on the predictor to have equal false positive rate^42^. To derive fair decisions with machine learning algorithms, three categories of approaches have been proposed to mitigate biases^41,43^: (1)*Pre-processing:* the original dataset is transformed so that the underlying discrimination towards some groups is removed^44^; (2) *In-processing:* either by adding a penalization term in the objective function^45^ or imposing a fairness-relevant constraint^46^; (3) *Post-processing:* further recompute the results from predictors to improve fairness^47^.

#### Bias and fairness in MIMIC-III

With clinical notes^48,49^ or temporal measurements^4,50,51^ or both^52^ from MIMIC-III considered, fairness evaluation and bias mitigation have been studied recently for tasks such as mortality prediction^4,48–52^, phenotyping^49,52^, readmission^50^, length of stay^51^, etc. To evaluate data and prediction fairness for the aforementioned healthcare tasks, attributes like ethnicity^4,48,49,51,52^, gender^49,51,52^, insurance^49,52^, age^48^ and language^49^, are considered most often to split patients into different protected groups.

When making medical decisions based on text data like clinical notes, word embeddings, used as machine learning inputs, have been demonstrated to propagate unwanted relationships with regard to different genders, language speakers, ethnicities, and insurance groups^49,52^. With respect to gender and insurance type, differences in accuracy and therefore machine bias has been observed for mortality prediction^50^. To mitigate biases and improve prediction fairness, Chen et al. argued that collecting data with adequate sample sizes and predictive variables measures is an effective approach to reduce discrimination without sacrificing accuracy^4^. Martinez et al. proposed an in-processing approach where the fairness problem is characterized as a multi-objective optimization task, where the risk for each protected group is a separate objective^48^. After well-trained machine learning models make predictions, equalized odds post-processing^52^ and updating predictions according to the weighted sum of utility and fairness^51^ were introduced respectively as effective post-processing approaches.

To continue the dataset bias and algorithmic fairness study on MIMIC-IV, we follow previous fairness study work and adopt the following commonly used demographic features as protected attributes: (1) *ethnicity*, (2) *gender*, (3) *marital status*, (4) *Age*, and (5) *insurance type*. For dataset bias, we analyze the average adoption and duration of five types of ventilation treatment on patients from different groups. For algorithmic fairness, we evaluate the performance of state-of-the-art machine learning approaches for mortality prediction in terms of accuracy and fairness.

### Interactions between interpretability and fairness

Besides accuracy, interpretability and fairness are two important aspects that businesses and researchers should take into consideration when designing, deploying, and maintaining machine learning models^53^.

It is well acknowledged that model interpretability methods, when applied to trained models, act as an important tool towards developing fairer ML systems^54^ since interpretations can help detecting and mitigating bias during data collection or labeling^55–57^. When the feature importance is leveraged to interpret model predictions, failure of fairness can be identified by detecting whether the feature has a larger effect than it should have^58,59^. For instance, Adebayo et al. show that gender is of low importance among all studied demographic features in a bank’s credit limit model, which indicates that the bank’s algorithm is not overly dependent on gender in making credit limit determinations^58^. Recently, connections between interpretability and fairness have been quantitatively studied by comparing fairness measures and feature importance measure: there is a direct relation between SHAP value difference and equality of opportunity after removing bias with reweighing techniques and measuring feature importance with SHAP on Adult, German, Default and COMPAS datasets^60^.

However, the effect of enhancing or enforcing one aspect of interpretability/fairness directly in machine learning models is relatively unexplored. Kleinberg et al.^61^ demonstrate a fundamental inconsistency between the model interpretability (measured as model simplicity) and fairness (equity): every simple prediction function can be outperformed by a more complex one with improved efficiency and equity. Jabbari et al.^62^ discovers several different types of trad-offs between interpretability and fairness. Enforcing fairness may also hinder the interpretability: Wang & Han et al.^63^ discuss that common approaches to enforcing fairness, including pre-processing of features and post-processing of predictions, involve non-interpretable manipulations and cannot be corrected for an interpretable model afterwards. In contrast, we leverage feature importance and interactions derived from both interaction and attribution approaches as tools to analyze models’ fairness and remove violating interactions within these models.

## MIMIC-IV dataset

In this section, we describe the following preprocessing steps of the MIMIC-IV dataset: cohort selection, feature selection, and data cleaning. We also report the distributions of demographic, admission and comorbidity variables within the dataset.

### Dataset description

MIMIC-IV^5,6^ is a publicly available database of patients admitted to the Beth Israel Deaconess Medical Center (BIDMC) in Boston, MA, USA. It contains de-identified data of 383,220 patients admitted to an intensive care unit (ICU) or the emergency department (ED) between 2008 and 2019. Till the day when we finished all experiments, the latest version of MIMIC-IV is v0.4 and only provides public access to the electronic health record data of 50,048 patients admitted to the ICU, which is sourced from the clinical information system MetaVision at the BIDMC. Therefore, we design the following data preprocessing procedures for the ICU data part of MIMIC-IV. [All methods were carried out in accordance with relevant guidelines and regulations] on this dataset.

### Preprocessing

#### Cohort selection

Following the common practice in^1,3^, we select ICU stays satisfying the following criteria as the cohort: (1) the patient is at least 15 years old at the time of ICU admission; (2) the ICU stay is the first known ICU stay of the patient; (3) the total duration of ICU stay is between 12 h and 10 days. After the cohort selection, we collect 45,768 ICU stays as the cohort. According to the cohort selection criterion (2), each ICU stay corresponds to one unique patient and one unique hospital admission.

#### Data cleaning and feature selection

We follow the same data cleaning procedure in^1^ to handle: (1) Inconsistent units. We convert features with multiple units to their major unit. (2) Multiple recordings at the same time. We use the average value for numerical features and the first appearing value for categorical features. (3) Range of feature values. We use the median of the range as the value of the feature.

We select 164 features from the following groups, a detailed list of all selected features is in Table 8 in Appendix:

- Electronic healthcare records (EHR). We modify the feature list used in^1^ and extract 122 features after removing features that are no longer available in MIMIC-IV.
- Demographic features. We extract 5 from patients’ demographic information.
- Admission features. We extract 4 from admission records.
- Comorbidity features. We extract binary flags of 33 types of comorbidity using patients’ ICD codes.

#### Data filtering, truncation, aggregation and imputation

Data Filtering After specifying the list of features, we further filter ICU stays from the cohort and only keep those that have records of selected EHR features for at least 24 h and at most 10 days, starting from the first record within 6 h prior to ICU admission time. We have 43005 ICU stays after the filtering. Other works^3^ extract the first 30-hour data and drop data from the last 6 h to avoid information leakage of positive mortality labels to features measured within 6 h prior to deathtime. We find that most (96.02%) of the patients with positive in-hospital mortality labels have measurements for over 30 h prior to their deathtime, thus we omit this processing step. Truncation For each ICU stay, we only keep the data of the first 24 h, starting from the first record within 6 h prior to its ICU admission time. Aggregation For each ICU stay, we aggregate its records hourly by taking the average of multiple records within the same hourly time window. Imputation We perform forward and backward imputation to fill missing values. For cases where certain features of some patients are completely missing, we fill with mean values of corresponding features in the training set.

### Dataset summary

After all preprocessing steps, we obtain features of the shape (*N*, *T*, *F*), where $N=43005$ is the number of ICU stays (data samples), $T=24$ is the number of time steps with 1-h step size, and $F=164$ is the total number of features. We also process the data into the tabular form $(N, F^\prime )$ by replacing sequential EHR features with the summary over time steps including minimum, maximum, and mean values (for the urinary_output_sum feature we have summation in addition), where $F^\prime = 409$. We show the distribution of demographic, admission, and comorbidity features grouped by patients’ in-hospital mortality status in Table 9 in Appendix. We also demonstrate differences between the preprocessed MIMIC-IV data in this work and the preprocessed MIMIC-III data from^1^ in Table 1.

**Table 1:** Differences between preprocessed MIMIC-III in^1^ and preprocessed MIMIC-IV.

	MIMIC-III	MIMIC-IV (this work)
# Samples	35,627	43,005
# Temporal features	135	122
# Demographic features	1	5
# Admission features	1	4
# Comorbidity features	3	33

## Interpretability evaluation

In this section, we evaluate the performance of various feature importance interpretability methods on multiple models for the in-hospital mortality prediction task. We describe the task, models, interpretability methods, and the evaluation method in detail and report the evaluation results.

### Task description

Mortality prediction is one primary outcome of high interest of hospital admissions, and is widely considered in other benchmark works^1–3,64^. We use the in-hospital mortality prediction task to train different models and evaluate the performance of various interpretability methods. We formulate the in-hospital mortality prediction task as a binary classification task. Given the observed sequence of features ${\varvec{X}}\in {\mathbb {R}}^{T\times F}$ of one patient (or its summary ${\varvec{x}}\in {\mathbb {R}}^{F}$, depending on the model), the model gives the probability that the patient dies during his/her hospital admission after being admitted to ICU. In MIMIC-IV, a patient has in-hospital mortality if and only if his/her deathtime exists in the mimic_core.admissions table. We randomly divide 60% data for training, 20% for validation and 20% for test.

### Models

We consider following models: (1) AutoInt^65^. A model that learns feature interaction automatically via self-attentive neural networks. (2) LSTM^66^. Long short-term memory recurrent neural network, which is a common baseline for sequence learning tasks. (3) TCN^67^. Temporal convolutional networks, which outperform canonical recurrent networks across various tasks and datasets. (4) Transformer^68^. A network architecture based solely on attention mechanisms. Here we only adopt its encoder part for the classification task. (5) IMVLSTM^69^. An interpretable model that jointly learns network parameters, variable and temporal importance, and gives inherent feature importance interpretation. We use sequence data as input for (2–5), and the summary of sequence data as input for (1) since AutoInt only processes tabular data in its original implementation.

We use the area under the precision-recall curve (AUPRC) and the area under the receiver operating characteristic curve (AUROC) as metrics for binary classification. The performance of all models considered in this work is shown in Table 2.

**Table 2:** Classification performance of all considered deep models.

AutoInt		LSTM		TCN		Transformer		IMVLSTM	
AUPRC	AUROC	AUPRC	AUROC	AUPRC	AUROC	AUPRC	AUROC	AUPRC	AUROC
0.508	0.901	0.660	0.938	0.666	0.928	0.686	0.939	0.769	0.955

### Interpretability methods

Interpretation of deep learning models is still a rapidly developing area and contains various aspects. In this work, we focus on the interpretation of feature importance, which estimates the importance of single features for a given model on a specific task. Estimation of feature importance helps improve the model, builds trust in prediction and isolates undesirable behavior^7^. Recent works^7,32,35^ have developed methods for evaluating feature performance estimation without access to the ground truth of feature importance, which fits scenarios in healthcare domains well: ground-truth feature importance for healthcare applications is either the problem we need to solve itself or requires extraction from a huge amount of domain knowledge. Therefore, we choose the interpretation of feature importance as the target aspect for evaluating interpretability methods.

Formally, given a function $M: {\mathbb {R}}^{d_{in}}\rightarrow {\mathbb {R}}^{d_{out}}$ and the input (flattened) feature vector ${\varvec{x}}\in {\mathbb {R}}^{d_{in}}$, the interpretation of feature importance gives a non-negative score ${\varvec{s}}({\varvec{x}})\in {\mathbb {R}}^{d_{in}}$, where $s({\varvec{x}})_i$ is the importance of $x_i$ to $M({\varvec{x}})$.

We select the following interpretability methods to compare their feature importance estimation results. Notice that some interpretability methods give signed scores (or “attributions”), where signs reflect positive/negative contributions of features to the output, and we use the absolute values of signed scores as importance scores. For methods requiring a baseline input vector, unless otherwise specified, we follow the method in^32^ and randomly sample ${\varvec{x}}^\prime \in {\mathbb {R}}^{d_{in}}$, where $x^\prime _i \sim {\mathcal {U}}[0, 1]$.

**(1) Gradient based methods**

- *Saliency*^9^ Saliency returns the gradients with respect to inputs as feature importance: ${\varvec{s}}({\varvec{x}}) = \frac{\partial M({\varvec{x}})}{\partial {\varvec{x}}}$. By taking the first-order Taylor expansion of the neural network at the input, $M({\varvec{x}})\approx (\frac{\partial M({\varvec{x}})}{\partial {\varvec{x}}})^{\intercal }{\varvec{x}}+ b$, which is a linear approximation of the network, the gradient $\frac{\partial M({\varvec{x}})}{\partial x_i}=s({\varvec{x}})_i$ is the coefficient of the *i*-th feature.
- *IntegratedGradients*^10^ IntegratedGradients assigns an importance score to each input feature by approximating the integral of gradients of the model’s output with respect to the inputs along the path (straight line) from given baselines
  1 $$\begin{aligned} \mathrm {IntegratedGradients}({\varvec{x}})_i = (x_i - x^\prime _i)\times \int _{\alpha =0}^1 \frac{\partial M({\varvec{x}}^\prime + \alpha ({\varvec{x}}- {\varvec{x}}^\prime ))}{\partial x_i} d\alpha , \end{aligned}$$
  where ${\varvec{x}}^\prime$ is the baseline.
- *DeepLift*^11,12^ DeepLift decomposes the output prediction of a neural network on a specific input by backpropagating the contributions of all neurons in the network to every feature of the input.
  2 $$\begin{aligned} \mathrm {DeepLift}({\varvec{x}})_i = (x_i - x^\prime _i) \times \frac{\partial ^g M({\varvec{x}})}{\partial x_i}, g(z_t) = \frac{f_t(z_t) - f_t(z_t^\prime )}{z_t - z_t^\prime }, \end{aligned}$$
  where $\frac{\partial ^g M({\varvec{x}})}{\partial x_i} = \sum _{p\in P_{io}}(\prod _{(s,t)\in p} w_{ts} \prod _{(s,t)\in p} g(z_t))$. $P_{io}$ is the set of all paths from the *i*-th input feature to the output neuron in the network. (*s*, *t*) is a pair of connected neurons in path *p*. Each neuron *t* contains a linear transformation $z_t = \sum _{q\in Pa(t)}w_{tq}o_q + b_t$ followed by a nonlinear mapping $o_t = f(z_t)$.
- *GradientShap*^13^ GradientShap approximates SHAP (SHapley Additive exPlanations) values by computing the expectations of gradients by randomly sampling from the distribution of baselines. It first adds white noise to each input sample and selects a random baseline from a given distribution, then selects a random point along the path between the baseline and the input with noise, and computes the gradient of outputs with respect to the random point. The procedure is repeated for multiple times to approximate the expected values of gradients $E(\frac{\partial M({\varvec{x}})}{\partial {\varvec{x}}})$. The final SHAP value for the *i*-th feature is $E(\frac{\partial M({\varvec{x}})}{\partial x_i}) \times (x_i - x^\prime _i)$.
- *DeepLiftShap*^13^ It extends DeepLift algorithm and approximates SHAP values using DeepLift. For each input, it samples baselines from a given distribution and computes the DeepLift score for each input-baseline pair and averages the resulting scores per input example as the output.
- *SaliencyNoiseTunnel*^14^ SaliencyNoiseTunnel adds Gaussian noise to the input sample and averages the calculated attributions using Saliency method as the output.

**(2) Perturbation based methods**

- *ShapleySampling*^15,16^ Shapley value gives attribution scores by taking each permutation of the input features and adding them one-by-one to a given value. Since the computation complexity is extremely high for large numbers of features, ShapleySampling takes some random permutations of the input features and averages the marginal contribution of features.
- *FeaturePermutation*^17^ FeaturePermutation permutes the input feature values randomly within a batch and computes the difference between original and shuffled outputs as the result.
- *FeatureAblation*^18^ FeatureAblation replaces each input feature with a given baseline value and computes the difference in output as the result.
- *Occlusion*^19^ Occlusion replaces each contiguous rectangular region with a given baseline and computing the difference in output as the result.
- *ArchDetect*^8^ It utilizes the discrete interpretation of partial derivatives. While the original paper considers both single features and feature pairs, we here only apply it to single features, since the evaluation method in this work is designed for single feature importance only. In the single feature case, the importance score of the *i*-th feature is
  3 $$\begin{aligned} \mathrm {ArchDetect}({\varvec{x}})_i = \left( \frac{M({\varvec{x}}_{\{i\}} + {\varvec{x}}^\prime _{\backslash \{i\}}) - M({\varvec{x}}^\prime _{\{i\}})}{x_i - x^\prime _i}\right) ^2, \text { where } ({\varvec{x}}_{{\mathcal {I}}})_i = \left\{ \begin{array}{cc} x_i , &{} ~\mathrm {if}~i\in {\mathcal {I}}; \\ 0 , &{} ~\mathrm {otherwise} . \\ \end{array} \right. \end{aligned}$$
  Here we select ${\varvec{x}}^\prime = {\varvec{0}}\in {\mathbb {R}}^{d_{in}}$.

**(3) Glassbox interpretation.** If the model’s architecture provides feature importance scores directly as a part of the output of the model, such as the attention score of each feature, we call this interpretation as “Glassbox” and regard it as an extra baseline.

**(4) Random baseline.** As a baseline, we randomly shuffle all features as the feature importance ranking.

For models in “Models” Section, AutoInt maps categorical features to embeddings using learnable dictionaries and has no gradient on categorical features, thus gradient based methods are not applicable. Only IMVLSTM model has Glassbox interpretation.

### Evaluation method

Since acquiring the ground-truth feature importance is challenging for mortality prediction tasks, we evaluate one feature importance estimation by gradually dropping most important features it gives at certain ratios from the dataset and observe the degradation of the model’s performance. The larger the degradation is, the better the estimation is, since it identifies the features most helpful for the model on the task.

More specifically, we use ROAR (remove and retrain) proposed in^7^ for evaluation. For each interpretability method, we replace the most important features of certain fractions of each data sample with a fixed uninformative value. We conduct this in both training and test sets. Then we retrain the model with the modified training set and evaluate its classification performance on the modified test set. By retraining the model on datasets with features removed, ROAR ensures that train and test data comes from a similar distribution and reduces the impact on the model’s performance of data distribution discrepancy, so that the degradation of performance is caused by the removal of information instead of the shift of data distribution.

For sequence input ${\varvec{X}}\in {\mathbb {R}}^{T\times F}$, we flatten it and give feature importance scores for all $T\times F$ features. For the *i*-th feature, we use its mean value in the training set as its uninformative value. We evaluate each interpretability method with feature drop ratios $10\%,20\%,\ldots ,100\%$ and plot the curve of model performance with respect to feature drop ratio for each model.

### Results

#### Evaluation of interpretability methods

**Figure 1:**
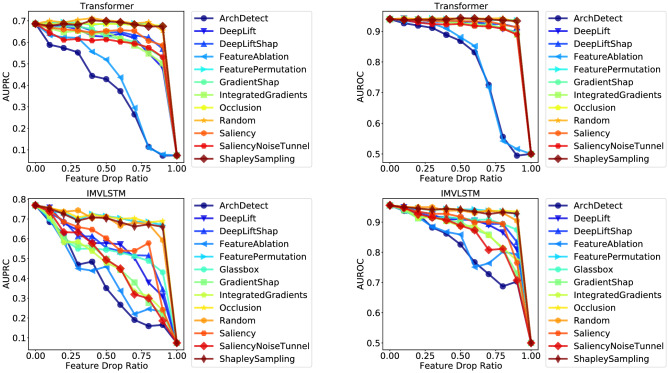
Curves of performance metric w.r.t feature drop ratio.

Figure 1 shows the curves of model performance (measured with AUPRC and AUROC respectively) with respect to the feature drop ratio of different interpretability methods for the top-2 models (Transformer & IMVLSTM), refer to Section 8.3 for all curves. Table 3 gives the quantitative results of area under the curve (AUC). A lower value of AUC means that the performance curve drops faster with the increase of feature drop ratio, thus indicates that the interpretability method gives a better ranking of feature importance.

**Table 3:** Area under the curve (AUC) of interpretability methods for each model and each classification performance metric evaluated using ROAR. AUC is measured for two prediction metrics (AURPC and AUROC) respectively. Lower AUC indicates more rapid prediction performance drop and better feature importance interpretation.

Interpreters	AutoInt		LSTM		TCN		Transformer		IMVLSTM	
	AUPRC	AUROC	AUPRC	AUROC	AUPRC	AUROC	AUPRC	AUROC	AUPRC	AUROC
Random	0.401	0.842	0.615	0.909	0.605	0.901	0.662	0.918	0.669	0.915
Glassbox	×	×	×	×	×	×	×	×	0.533	0.892
Saliency	×	×	0.558	0.898	0.587	0.893	0.616	0.909	0.566	0.884
IntegratedGradients	×	×	0.586	0.899	0.593	0.899	0.588	0.903	0.465	0.863
DeepLift	×	×	0.575	0.900	0.598	0.898	0.594	0.905	0.542	0.883
GradientShap	×	×	0.561	0.893	0.592	0.899	0.600	0.904	0.470	0.858
DeepLiftShap	×	×	0.569	0.897	0.607	0.901	0.619	0.909	0.554	0.887
SaliencyNoiseTunnel	×	×	0.551	0.892	0.581	0.896	0.578	0.899	0.475	0.851
ShapleySampling	0.456	0.866	0.628	0.910	0.613	0.898	0.655	0.916	0.668	0.917
FeaturePermutation	0.454	0.866	0.624	0.910	0.616	0.903	0.655	0.917	0.677	0.918
FeatureAblation	0.279	0.733	0.438	0.811	0.479	0.824	0.425	0.792	0.408	0.830
Occlusion	0.456	0.866	0.617	0.909	0.609	0.898	0.653	0.917	0.684	0.920
ArchDetect	0.251	0.696	0.369	0.774	0.446	0.818	0.379	0.784	0.382	0.805

We have the following observations: (1) ArchDetect gives the best performing feature importance estimation overall. From Fig. 1, we observe that the curve of ArchDetect drops the fastest for all models on both metrics. Quantitative results in Table 3 also show that ArchDetect has the lowest AUC. Therefore, for the in-hospital mortality task, the feature importance ranking given by ArchDetect is the most reasonable one among results of all interpretability methods considered in this work. (2) Gradient based methods perform well on LSTM, Transformer and IMVLSTM models, but are no better than a random guess on TCN. AUC of both metrics of gradient based methods is significantly lower than that of random guessing for LSTM, Transformer and IMVLSTM. But for TCN, even the best performing gradient based method SaliencyNoiseTunnel has AUC close to random guessing (0.581 vs. 0.605 for AUPRC and 0.896 vs. 0.901 for AUROC). (3) Attention scores are not necessarily the best estimation of feature importance. In IMVLSTM, the Glassbox baseline utilizes attention scores the model gives as an estimation of feature importance. Although it outperforms the random guessing baseline, it is not among the best interpretation methods and is inferior to methods such as ArchDetect and IntegratedGradients. Similar observations also exist in the natural language processing domain^70,71^, where attention weights largely do not correlate with feature importance.

#### Identified important features

**Figure 2:**
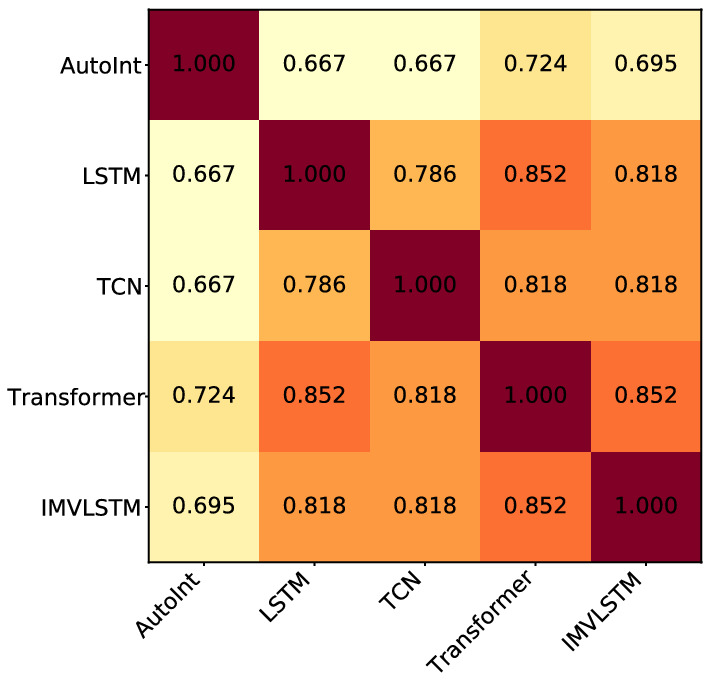
Jaccard similarity of top-50 most important features identified in all models.

*Similarity of Important Features from Different Models* We further investigate and compare important features given by different prediction models with the best performing interpretablity method ArchDetect in  “Results” Section for a qualitative evaluation of its effectiveness. Since ArchDetect gives local feature importance for each data sample respectively, we aggregate local results for a global qualitative evaluation with following steps: (1) for each sample, get the rank of importance for each feature; (2) calculate the average of ranks for each feature over all data samples; (3) sort the averaged ranks of features from (2) as the global ordering of importance for all features. We then verify the effectiveness of feature importance estimation given by ArchDetect from following aspects:

*Similarity of important features from different models* Figure 2 shows the Jaccard similarity of top-50 most important features identified in models. We observe that (1) the Jaccard similarity of top-50 most important features from any pair of two models is above 0.667; (2) each pair of models accepting sequential data (LSTM, TCN, Transformer, and IMVLSTM) has a Jaccard similarity over 0.786. This result demonstrates that ArchDetect identifies similar sets of important features when applied to various models, which is necessary for its correctness since the ground truth set of important features is unique.

*Overlapping and disagreement of feature importance across models* Since IMV-LSTM achieves the best mortality prediction performance, to take a closer look into the overlapping and disagreement of feature importance across models, we show differences of top-50 important features in other models from those in IMVLSTM in Table 4.

We observe several common anomalies across models: (1) Top-50 important features of AutoInt has larger discrepancy than others with those of IMVLSTM, which is coherent to its larger performance gaps in Table 2. (2) Importance of some comorbidities (features with indices falling into 123–125 and 134–163) tend to be overestimated by suboptimal models, while the importance of peripheral vascular (feature 138) is underestimated in AutoInt and LSTM. (3) Suboptimal models underestimate various labevent and chartevent features, and LACTATE (feature 57) is lacking in important features identified in each of them.

**Table 4:** Comparison of top-50 important features between suboptimal models and the best performing one.Importance of some comorbidities tend to be overestimated by suboptimal models, while the importance of peripheral vascular is underestimated in AutoInt and LSTM. Suboptimal models underestimate various labevent and chartevent features.

Model	Extra (identified in model but not in IMVLSTM)	Lacking (identified in IMVLSTM but not in model)
AutoInt	88. systolic_blood_pressure_abp_mean 90. body_temperature 134. congestive_heart_failure 135. cardiac_arrhythmias 140. paralysis 152.solid_tumor 153. rheumatoid_arthritis 160.alcohol_abuse 163. depression	31. HEMOGLOBIN 33. MCH 44. INR(PT) 45. PT 57. LACTATE 76. O2Flow 82. SpO2DesatLimit 91. pao2 138. peripheral_vascular
LSTM	32. MCHC 135. cardiac_arrhythmias 136. valvular_disease 143. diabetes_uncomplicated 146. renal_failure	39. CREATININE 57. LACTATE 82. SpO2DesatLimit 131. language 138. peripheral_vascular
TCN	77. Glucosefingerstick 134. congestive_heart_failure 136. valvular_disease 151. metastatic_cancer 152. solid_tumor	39. CREATININE 57. LACTATE 82. SpO2DesatLimit 93. urinary_output_sum
Transformer	135. cardiac_arrhythmias 136. valvular_disease 143. diabetes_uncomplicated 152. solid_tumor	45. PT 57. LACTATE 82. SpO2DesatLimit 124. HEM

*Visualization of Global Feature Importance Ranks and Comparison with Domain Knowledge* With the aim to (1) verify feature importance estimation results with domain knowledge, and (2) give an intuitive explanation of what features are important for mortality prediction task, we compare feature importance results given by ArchDetect with existing domain knowledge. More specifically, we collect features used for calculating 6 types of scores measuring the severity of illnesses that are supported by MIMIC-IV (https://github.com/MIT-LCP/mimic-code/tree/main/mimic-iv/concepts/score) and consider their union as important features for predicting mortality in domain knowledge. Scores include: Acute Physiology Score III (APS III)^72^, Logistic Organ Dysfunction Score (LODS)^73^, Oxford Acute Severity of Illness Score (OASIS)^74^, Simplified Acute Physiology Score II (SAPS II)^75^, Systemic inflammatory response syndrome (SIRS)^76^, Sequential Organ Failure Assessment (SOFA)^77^. We provide visualizations for all features in Figs. 9, 10, 11, 12, 13 and 14 in Appendix.

**Figure 3:**
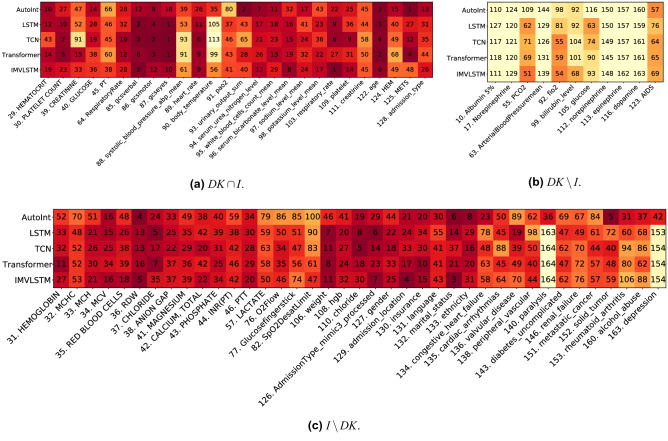
Visualization of global feature importance ranks for the overlapping and non-overlapping features between domain knowledge (*DK*) and interpretation results (*I*).

Denote as *DK* the union of features for calculating severity scores, and *I* the union of top-50 most important features identified by ArchDetect in all models. We visualize the global feature importance ranks given by ArchDetect for their overlapping parts ($DK\cap I$) and non-overlapping parts ($DK\setminus I$ and $I\setminus DK$) in Fig. 3. We observe that: (1) Most (70.3%, 26 out of 37) features considered important for mortality prediction in domain knowledge are also identified as important features via ArchDetect. (2) 11 features important in domain knowledge are not identified as important by ArchDetect (Fig. 3b). Instead, ArchDetect recognizes 39 features that are not covered by domain knowledge (Fig. 3c), which mainly includes labevent features (laboratory based measurements of fluid of the patient’s body, feature 31–46 and 108–110), respiratory-related features (76), comorbidity features (123–125 and 134–163), and demographic features (127–133). Such mismatches may provide useful insights for doctors and experts of better evaluations of mortality. (3) We notice that demographic features play important roles in prediction models, which may raise the concern of fairness. Thus we further investigate the fairness of data and models in the following section.

### Connection between interpretability and trustworthiness of deep learning models

Besides understanding the reasons or insights behind particular predictions made from deep learning models, how much we can trust these predictions also deserves serious consideration, especially when making critical decisions accordingly in domains such as autonomous driving, finance, and healthcare, etc. In order to study the connection between model interpretability and trustworthiness of deep learning models for our mortality prediction task, we are interested in how the trustworthiness of deep predictive models varies when the given features are removed step by step according to the recognized importance from diverse interpretability models.

Firstly, we quantify the model trustworthiness following the metric *NetTrustScore* $T_M$ defined in^78,79^:

$$\begin{aligned} Q_z(x,y)&={\left\{ \begin{array}{ll} C(y|x)^{\alpha },&{}\text {if }x\in R_{y=z|M}\\ (1-C(y|x))^{\beta },&{}\text {if }x\in R_{y\ne z|M} \end{array}\right. },\\ T_M(z)&=\int \int P(x,y)Q_z(x,y)dydx,\\ T_M&=\int P(z)T_M(z)dz, \end{aligned}$$

where *x* is the features that deep neural networks make predictions with, *y* and *z* are the predicted and true class label respectively, *C*(*y*|*x*) is the confidence represented by softmax outputs associated with the predicted class labels, $\alpha$ and $\beta$ denotes reward and penalty relaxation coefficients, $R_{y=z|M}$ denote the scenario that the prediction *y* from model *M* matches the oracle label *z*, *P*(*x*, *y*) is the probability of the occurrence of the sample (*x*, *y*), *P*(*z*) is the probability of occurrence for ground-truth label *z*.

**Figure 4:**
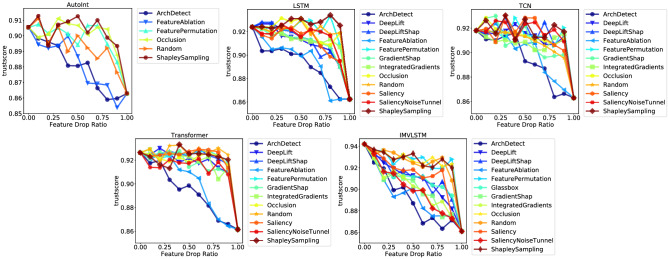
Curves of trustworthiness metric w.r.t feature drop ratio.

For mortality prediction, we set $\alpha =1$ and $\beta =1$ to penalize undeserved overconfidence and reward well-placed confidence equally. Therefore, the trustworthiness of binary classification models is quantified by $T_M=\frac{1}{|{\mathcal {D}}|}\sum _{(x,z)\in {\mathcal {D}}}C(z|x)$, where ${\mathcal {D}}$ is the testing dataset. We illustrate the variation of trustworthiness along with the feature drop ratio in Fig. 4, where the feature importance is computed by different interpretability methods on different predictive models. Considering the fact that predictive models fed with less task-relevant information will lead to less trustworthy predictions, we also include the way of randomly removing features as a baseline to alleviate the influence of information loss. Regardless of the underlying predictive models, we observe that if we remove the features with high importance computed by *ArchDetect* or *FeatureAblation*, the trustworthiness of all studied deep learning models drops massively compared with other interpretability methods as well as the random strategy. To quantify the connection between the feature importance provided by interpretability model and the trustworthiness of predictive models, we calculate the AUC of Fig. 4 and list the results in Table 5. We find that the two interpretability methods, *ArchDetect* and *FeatureAblation*, have consistently lower AUC values compared with the random feature removal strategy. This is consistent with our prior visual analysis that important features recognized by *ArchDetect* and *FeatureAblation* contribute a lot to the trustworthiness of predictions from deep learning models.

**Table 5:** Area under the curve (AUC) of interpretability methods for each model and the model trustworthiness metric evaluated using ROAR. Lower AUC indicates more rapid trustworthiness drop.

Interpreters	AutoInt	LSTM	TCN	Transformer	IMVLSTM
Random	0.893	0.919	0.908	0.924	0.924
Glassbox	×	×	×	×	0.910
Saliency	×	0.911	0.914	0.922	0.914
IntegratedGradients	×	0.912	0.907	0.918	0.902
DeepLift	×	0.910	0.912	0.918	0.909
GradientShap	×	0.910	0.908	0.916	0.901
DeepLiftShap	×	0.911	0.914	0.921	0.911
SaliencyNoiseTunnel	×	0.915	0.916	0.913	0.900
ShapleySampling	0.902	0.924	0.917	0.920	0.925
FeaturePermutation	0.898	0.920	0.913	0.921	0.925
FeatureAblation	0.881	0.894	0.900	0.901	0.894
Occlusion	0.901	0.919	0.912	0.919	0.926
ArchDetect	**0.880**	**0.892**	**0.894**	**0.894**	**0.891**

Lower is better. Best values are in [bold].

## Fairness evaluation

In this section, we first describe the set of demographic features considered as protected attributes. We then investigate the extent of which disparate treatment exists within the MIMIC-IV dataset. Given that the in-hospital mortality predictors can be further utilized in a down-stream decision-making policy, we audit their fairness across various protected attributes.

### Protected attributes

MIMIC-IV came with a set of demographic features that are helpful for the task of auditing in-hospital mortality predictors for prediction fairness. Protected classes under the Equal Credit Opportunity Act (ECOA) include the following: *age, color, marital status, national origin, race, recipient of public assistance, religion, sex*^80^. For our task, we consider a subset of such protected classes available within the dataset. To remove uncertainty within our analysis, we further identify and drop examples with unclear attributes, such as ‘None’, ‘Unknown’, or ‘Unable to obtain’. Table 6 lists the attributes and subgroups used within our analysis. Note that *age* is grouped by quartiles. Refer to Table 9 in the Appendix for more information on each subgroup.

**Table 6:** Protected attributes and subgroups within MIMIC-IV.

Protected attributes	Groups
Ethnicity	[‘ASIAN’, ‘BLACK/AFRICAN AMERICAN’, ‘HISPANIC/LATINO’, ‘OTHER’, ‘WHITE’]
Gender	[‘FEMALE’, ‘MALE’]
Marital status	[‘MARRIED’, ‘SINGLE’, ‘DIVORCED/WIDOWED’]
Age	[‘<55 YRS’, ‘55-67 YRS’, ‘67-78 YRS’, ‘>=78 YRS’]
Insurance	[‘MEDICAID/MEDICARE’, ‘PRIVATE’]

### Fair treatment analysis

Disparate treatment is unlawful discrimination in US labor law. Title VII of the United States Civil Rights Act is created to prevent unequal treatment or behavior toward someone because of a protected attribute (e.g. race, gender, or religious beliefs). Although the type and duration of treatment received by patients are determined by multiple factors, analyzing treatment disparities in MIMIC-IV can give us insights in potential biases in treatment received by different groups. Previously, there have been a few works pointing out the racial disparities in end-of-life care between cohorts of black and white patients within MIMIC-III^81,82^. In a similar spirit, we additionally investigate treatment adoptions and duration across not only ethnicity, but also gender, age, marital status, and insurance type.

#### Evaluation method

In MIMIC-IV, 5 categories of mechanical ventilation received by patients have been recorded: HighFlow, InvasiveVent, NonInvasiveVent, Oxygen, and Trach. We first extract the treatment duration and then label the patients with no record as no intervention adoption. If a patient had multiple spans, such as an intubation-extubation-reintubation, then we consider the patient’s treatment duration to be the sum of the individual spans.

#### Results

**Figure 5:**
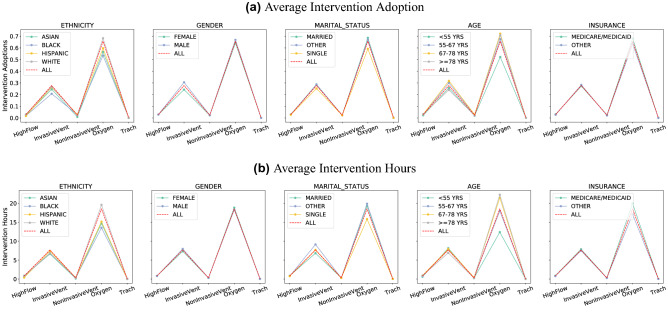
Average adoption and hours of intervention in general and in subjects from different groups.

Figure 5 plots the intervention adoption rate and intervention duration across different protected attributes. We observe that: (1) There exists disparate treatments, which is most evident across different ethnic groups. The first column in Fig. 5 indicates that on average the Black cohort is less likely to receive ventilation treatments, while also receiving a shorter treatment duration. Similar observations that people from different racial groups tend to receive different treatment^83,84^ or health care plans^81,82^ have been reported in literature. Similarly, this is also observed across groups split by marital status, where single patients tend to receive shorter and fewer ventilation treatments as opposed to married patients, and similarly with patients with public or private insurances. (2) There are numerous hidden confounders in analyzing disparate treatment. The fourth column in Fig. 5 indicates more treatments provided to older patients. However, one can imagine that cause of this is medically relevant as the older cohort tends to require more care. Similarly, patients with generous public insurance can more easily afford more treatments. In particular, we note that it is difficult to precisely determine whether the differences in treatment are due to intentional discrimination or differences caused by other confounders. At the current junction, we suspect a closer look at causal analysis in future works can help address this problem.

### Fair prediction analysis

Fairness in machine learning is a rapidly developing field with numerous definitions and metrics for prediction fairness with respect to two notions: individual and group fairness. For our binary classification task of in-hospital mortality prediction, we consider the group notion where a small number of protected demographic groups *G* (such as racial groups) is fixed, and we then ask for the classification parity of certain statistics across all of these protected groups.

#### Fairness metrics

Most recently, a multitude of statistical measures have been introduced for group fairness, most notable are statistics that ask for the equality of the false positive or negative rates across all groups *G* (often known as *‘equal opportunity’*^42^) or the equality of classification rates (also known as *statistical parity*). Interestingly, it has been proven that some of the competing definitions and statistics previously proposed are mutually exclusive^85^. Thus, it is impossible to satisfy all of these fairness constraints.

In our case, it is often necessary for mortality assessment algorithms to explicitly consider health-related protected characteristics, especially the age of the patients. Hence, an age-neutral assessment score can systematically overestimate a young person’s mortality risk, and can in turn encourage unnecessarily medical interventions. Similarly, enforcing equality of mortality classification rates can likewise lead to discriminatory decision making. Hence, we choose AUC (area under the ROC curve) as our evaluation metrics to audit fairness across subgroups. First, it encompasses both FPR and FNR, which touches on the notion of equalized opportunity and equalized odds. Second, it is robust to class imbalance, which is especially important in the task of mortality prediction where mortality rates are $\sim 7\%$, Lastly, AUC is threshold agnostic, which does not necessitate setting a specific threshold for binary prediction that is used across all groups.

#### Evaluation method

To evaluate fairness on the MIMIC-IV dataset, we stratify the test set by groups (Table 6), and compute the model’s AUC for each protected group, similarly to^86^. In addition, we also added a stratification for the patient group with the largest common comorbidity, with HEM/METS for patients with lymphoma, leukemia, multiple myeloma, and metastatic cancer. We report (1) AUC(min): minimum AUC over all protected groups, (2) AUC(macro-avg): macro-average over all protected group AUCs and (3) AUC(minority): AUC reported for the smallest protected group in the dataset. Higher AUC is better for all three metrics.

Additionally, as MIMIC-IV is an ongoing data collection effort, we also investigate the relationships between the predictive performance of the mortality predictors and the data distribution with respect to each protected group. It was shown in^87^ that if the risk distributions of protected groups in general differ, such as mortality rates, threshold-based decisions will typically yield error metrics that also differ by group. Hence, we are interested in studying the potential source of the bias/differences in predictive performances from the MIMIC-IV training set.

#### Results

Figure 15 shows the training data distribution, mortality rates, and testing AUCs across each protected attribute for all patients and patients with HEM/METS, summarized over all five classifiers: AutoInt, LSTM, IMV-LSTM, TCN, and Transformer. Smaller gaps in AUC indicate equality in predictive performances, and larger gaps indicate potential inequalities. Table 7 gives the quantitative results of the area under the curve (AUC). Higher values of AUCs for each of the min, avg, and minority AUC metrics indicate better predictive performance with respect to the protected groups.

**Table 7:** Summarized Area under the curve (AUC) performance of the in-hospital mortality predictors evaluated on sets of protected groups. Higher AUC indicates better predictive performance.

Methods	Patient group	AUC overall	Minimum AUC over all protected groups	Macro-average AUC over all protected group	AUC For the smallest protected group
AutoInt	All	0.900	0.832	0.897	0.882
LSTM		0.941	0.896	0.939	0.932
TCN		0.937	0.883	0.936	0.948
Transformer		0.941	0.898	0.939	0.953
IMV-LSTM		0.955	0.918	0.954	0.968
AutoInt	HEM, METS	0.795	0.546	0.783	0.546
LSTM		0.842	0.726	0.830	0.777
TCN		0.832	0.696	0.822	0.696
Transformer		0.839	0.778	0.830	0.823
IMV-LSTM		0.884	0.845	0.879	0.862

We have the following observations: (1) IMV-LSTM performs the best overall on fairness measure with respect to AUC across different protected groups. Quantitatively, from Table 7, it is clear that IMV-LSTM has the highest AUC for both overall samples and the subgroups. We see that the minimum AUC for the protected subgroups is highest among the methods considered in this work. This indicates a higher lower bound over all protected attributes. Moreover, the AUC gap for minimum over protected groups is much larger than the next best model, Transformer, for the patient groups with HEM, and METS. (2) The in-hospital mortality predictors are in general fair, but less so for the subgroup of patients with the comorbidity HEM/METS. From Fig. 15, we observe that the maximum AUC gap across all attributes is at most 0.08, which is smaller than the maximum AUC gap for patients with HEM and METS at 0.11. The difference is more pronounced in the Ethnicity class, but can similarly be observed for other protected classes. In general, we note that all models are quite fair across ethnic groups, with small deviations in gender, and patient’s insurance. Across both sets of patients, we see that all classifiers are in general more accurate for younger patients ($<55$ years) versus older patients. (3) There exists a strong correlation between mortality rates and AUCs for each of the protected attributes. We observe that there is a strong correlation between group mortality rates and group AUC, with Pearson’s r = − 0.922 and a *p*-value < 0.00001. This shows that groups with higher mortality rates indicate lower AUC scores. From Fig. 15, we also observe that data with imbalanced representation between each subgroup does not impact predictive performance substantively.

## Interactions between interpretability and fairness

Fairness and interpretability are two critical pillars of the recent push for fairness, accountability, and transparency within deep learning. Overall, most interpretability works concern with explaining how the input features impact the final prediction, whether through feature importance or attributions, interactions, and knowledge distillation. Fairness on the other hand considers fairness metrics, optimization for fairness constraints, and the trade-off between accuracy and fairness. However, to the best of our knowledge, few work attempts to answer the question of how can interpretability help with fairness. What can we learn from our interpretability methods that would indicate either algorithmic bias or representation bias? In this section, we present concrete evidence to establish the initial connection between the two areas, but admittedly leave the fully investigation on the strength of this interaction for future work.

### Feature importance correlation with fairness metrics

**Figure 6:**
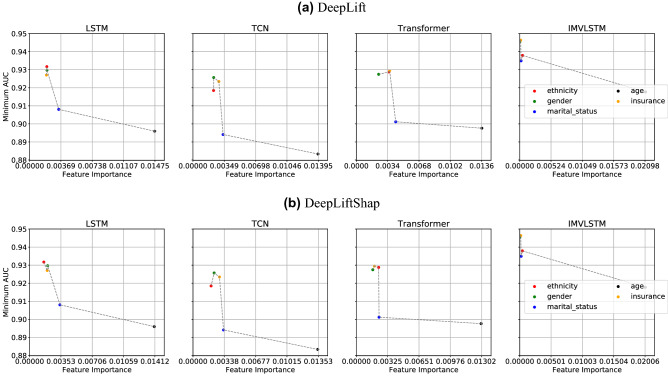
Interactions between *Feature Importance* from two interpretability approaches and fairness evaluation value *Min AUC* based on mortality predictions from four models.

Given mortality predictions made by state-of-the-art models on MIMIC-IV, we study the connections between feature importance induced by different interpretation approaches and the fairness measures in Fig. 6. For all the five protected attributes, we compute their respective feature importance by averaging the values produced from interpretability models across time and patients. Taking the feature importance as *x* axis and the minimum AUC from subgroups split by protected attributes as *y* axis, we are expecting to see a decreasing trend, where more important features have a higher possibility to lead to performance divergence in the split subgroups. We observe the expected trend consistently among all prediction models, when the interpretability approach *DeepLift* and *DeepLiftShap* are utilized. As shown in Fig. 6, age (black dot) is the most important feature compared with other protected attributes and the accuracy difference between young and old is more obvious than other group divisions. Similarly, ethnicity (red dot) and gender (green dot) are the least important features, which leads to much higher minimum AUC than other protected attributes. We plotted but did not observe obvious connections between feature importance from other interpretability approaches and other two fairness evaluation metrics.

### Feature importance scores across protected attributes

**Figure 7:**
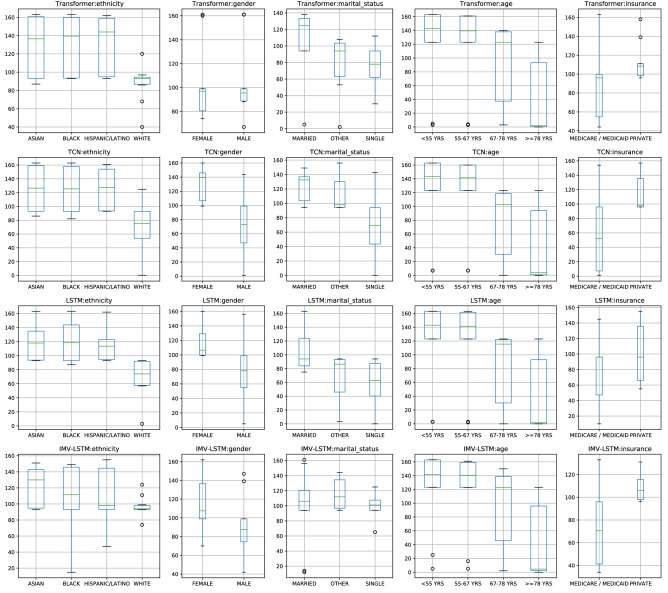
Feature rankings for each demographic feature for the four models: Transformer, TCN, LSTM, and IMV-LSTM, and each of the 12 interpretability methods: ArchDetect, DeepLiftShap, FeaturePermutation, IntegratedGradients, SaliencyNoiseTunnel, DeepLift, FeatureAblation, GradientShap, Occlusion, Saliency, and ShapleySampling.

Interpretability often concerns with *global* feature importance for the entire model and *local* feature importance for an individual sample with respect to its prediction. Here, we consider the group feature importance that builds upon *local* feature importance. Ideally, we want to measure how important each feature is across different groups with certain protected attributes. Hence, we define the group feature importance $g_i$ for feature *i* and protected attribute *A*, $g_{i,A} = \frac{1}{N_A}\sum _{j=1}^{N_A} \phi ^j_i,$ where $N_A$ is the size of the group with attribute *A*, and $\phi ^j_i$ is the *local* feature importance of the feature *i* for a person *j* with attribute *A*. The parity between $g_{i,A}$ would indicate a parity in how each feature is being used for different groups within a certain class of protected attributes. In the MIMIC-IV setting, we are interested in the importance of each of the demographic features used for the in-mortality prediction across the protected subgroups.

Since the scales of the feature importance scores are different for each interpretability method, we calculate the group feature importance for each demographic feature and rank their importance relative to other features within each interpretability method. Since feature importance is provided for {each hour timestep} x {each feature} within the first 24 h in the ICU, for all models, we additionally average the feature importance across timesteps. Figure 7 presents the box plot of the feature rankings for each demographic feature for the four models: Transformer, TCN, LSTM, and IMV-LSTM, and each of the 12 interpretability methods: ArchDetect, DeepLiftShap, FeaturePermutation, IntegratedGradients, SaliencyNoiseTunnel, DeepLift, FeatureAblation, GradientShap, Saliency, and ShapleySampling. A lower ranking indicates higher feature importance.

We observe that similar trends exist across different models of varying architectures, where a demographic feature is more important (has lower ranking) for specific groups. Out of 164 features used for each timestep, the feature ethnicity has the highest feature importance for the WHITE patients, similarly for the MALE patients with the feature gender, and the age group >= 78 YRS with the feature age, and so on. The protected attribute age is the most intuitive in this setting, where in-hospital mortality predictors would attribute high importance to elderly patients since that is a strong signal for mortality prediction. A similar case can be made the feature insurance, as patients with Medicare are often elderly. However, it is less intuitive for the ethnicity feature, as to why one subgroup would use the ethnicity feature more strongly than the other subgroups. This stark parity exists for all models, even for different methods of interpretability to obtain feature importance. In summary, we do note that feature importance, especially when viewed as group importance, can concretely reveal how a feature is being used for different groups. However, it is difficult to identify the confounders or features that strongly correlate with the ethnicity feature. Therefore we leave further study from causal perspectives for future work.

## Summary

*Limitations* Though we attempt to comprehensively evaluate the interpretability and fairness of deep learning models on MIMIC-IV, our works are not without its limitations. For evaluation of interpretability techniques, we examine the feature importance against domain knowledge from SAPS-II, which is a common but coarse patient severity score used by experts. However, the evaluation of feature importance can benefit from a more labelled healthcare dataset with known ground truth on feature importance ranks. For evaluation of fairness, we look at how sensitive features can influence both a model’s feature importance as well as hospital interventions. Although we touch on the interaction between interpretability and fairness in this work, future work using medical knowledge on causal influence will allow key insights into existing biases throughout healthcare applications.

*Conclusion* In this work, we conduct analysis on the MIMIC-IV dataset and several deep learning models in terms of model interpretability, dataset bias, algorithmic fairness, and the interaction between interpretability and fairness. We present quantitative evaluations of interpretability methods on deep learning models for mortality prediction, demonstrate the dataset bias in treatment in MIMIC-IV, verify the fairness of studied mortality prediction models, and reveal the disparities of feature importance among demographic subgroups.

## Figures and Tables

**Figure 8:**
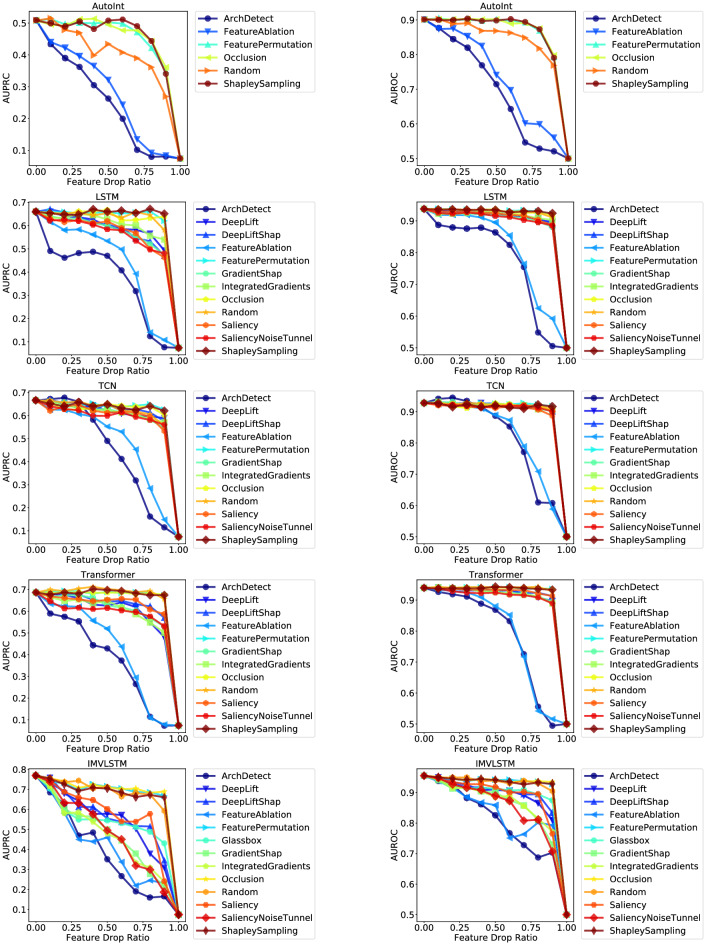
Curves of performance metric w.r.t feature drop ratio.

**Figure 9:**
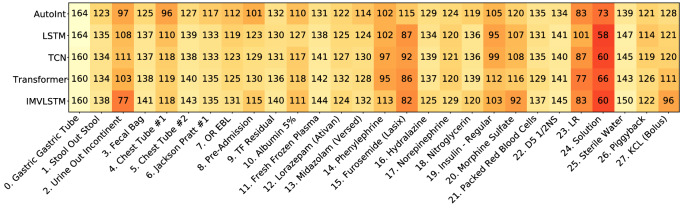
Visualization of global feature importance ranks (feature 0-27).

**Figure 10:**
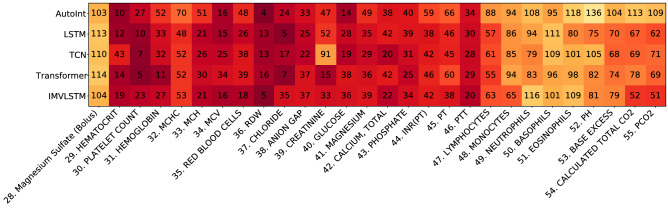
Visualization of global feature importance ranks (feature 28-55).

**Figure 11:**
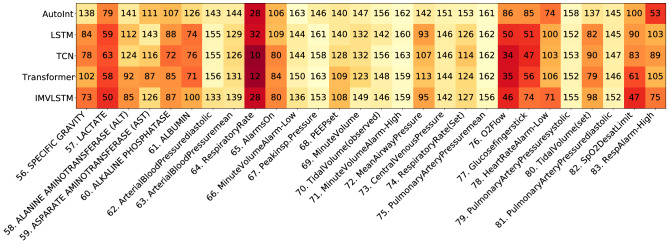
Visualization of global feature importance ranks (feature 56-83).

**Figure 12:**
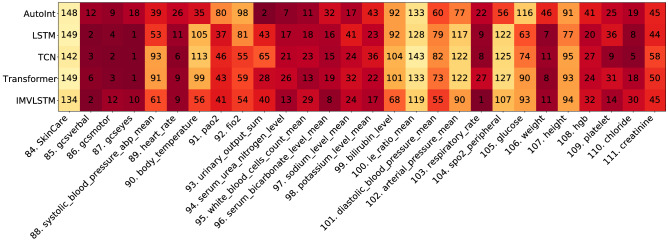
Visualization of global feature importance ranks (feature 84-111).

**Figure 13:**
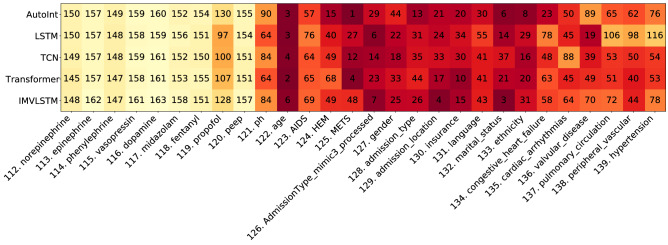
Visualization of global feature importance ranks (feature 112-139).

**Figure 14:**
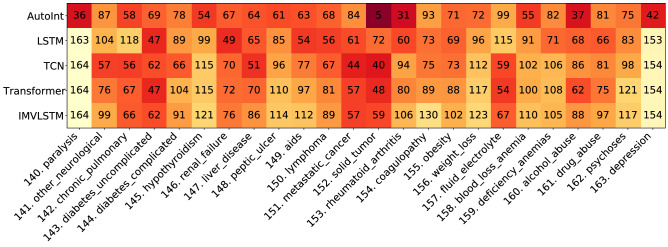
Visualization of global feature importance ranks (feature 140-163).

**Figure 15:**
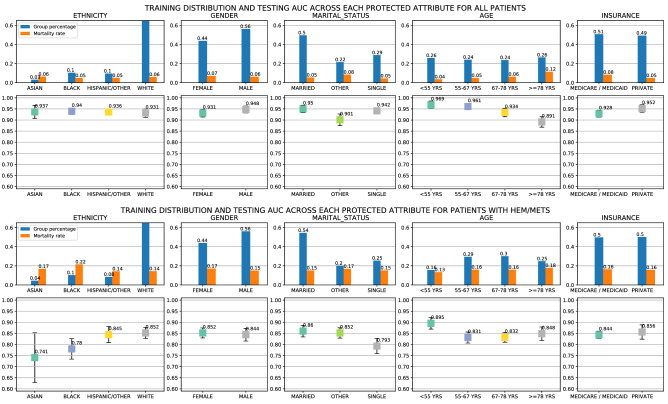
Training data distribution, mortality rates, and testing AUCs across each protected attribute for all patients and patients with HEM/METS, summarized over all five classifiers: AutoInt, LSTM, IMV-LSTM, TCN, and Transformer.

**Figure 16:**
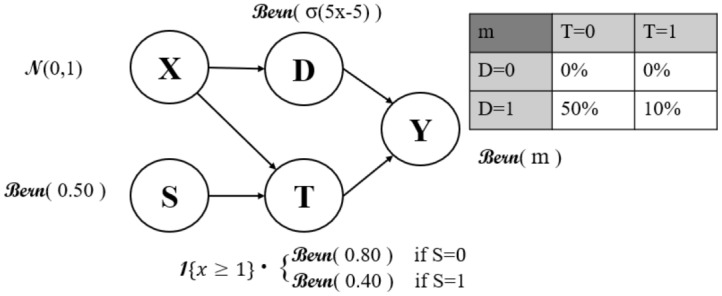
Data model predictions for the synthetic medical dataset with a biased latent treatment variable.

**Figure 17:**
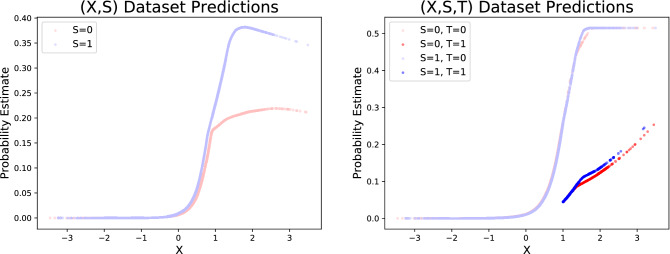
Model predictions for all patients (dots) in the synthetic medical dataset. S represents a sensitive attribute like Skin color. When the treatment T is biased on S, we observe how much the model relies on S for prediction (left). When source of bias is appropriately included in the model, we can both confirm the source of bias and remove the dependency on sensitive attributes from the predictive models (right).

## Data Availability

Figures are created using Matplotlib^88^ under the free software PSF license.

## Appendix

### List of Features

See Table 8.

**Table 8:** Full list of selected 164 features.

*i*-th feature	Feature name	Group	Tablename
0	Gastric gastric tube	EHR	mimic_icu.outputevents
1	Stool out stool	EHR	mimic_icu.outputevents
2	Urine out incontinent	EHR	mimic_icu.outputevents
3	Fecal bag	EHR	mimic_icu.outputevents
4	Chest tube #1	EHR	mimic_icu.outputevents
5	Chest tube #2	EHR	mimic_icu.outputevents
6	Jackson pratt #1	EHR	mimic_icu.outputevents
7	OR EBL	EHR	mimic_icu.outputevents
8	Pre-admission	EHR	mimic_icu.outputevents
9	TF residual	EHR	mimic_icu.outputevents
10	Albumin 5%	EHR	mimic_icu.inputevents
11	Fresh frozen plasma	EHR	mimic_icu.inputevents
12	Lorazepam (Ativan)	EHR	mimic_icu.inputevents
13	Midazolam (Versed)	EHR	mimic_icu.inputevents
14	Phenylephrine	EHR	mimic_icu.inputevents
15	Furosemide (Lasix)	EHR	mimic_icu.inputevents
16	Hydralazine	EHR	mimic_icu.inputevents
17	Norepinephrine	EHR	mimic_icu.inputevents
18	Nitroglycerin	EHR	mimic_icu.inputevents
19	Insulin - regular	EHR	mimic_icu.inputevents
20	Morphine sulfate	EHR	mimic_icu.inputevents
21	Packed red blood cells	EHR	mimic_icu.inputevents
22	D5 1/2NS	EHR	mimic_icu.inputevents
23	LR	EHR	mimic_icu.inputevents
24	Solution	EHR	mimic_icu.inputevents
25	Sterile water	EHR	mimic_icu.inputevents
26	Piggyback	EHR	mimic_icu.inputevents
27	KCL (Bolus)	EHR	mimic_icu.inputevents
28	Magnesium sulfate (Bolus)	EHR	mimic_icu.inputevents
29	HEMATOCRIT	EHR	mimic_hosp.labevents
30	PLATELET COUNT	EHR	mimic_hosp.labevents
31	HEMOGLOBIN	EHR	mimic_hosp.labevents
32	MCHC	EHR	mimic_hosp.labevents
33	MCH	EHR	mimic_hosp.labevents
34	MCV	EHR	mimic_hosp.labevents
35	RED BLOOD CELLS	EHR	mimic_hosp.labevents
36	RDW	EHR	mimic_hosp.labevents
37	CHLORIDE	EHR	mimic_hosp.labevents
38	ANION GAP	EHR	mimic_hosp.labevents
39	CREATININE	EHR	mimic_hosp.labevents
40	GLUCOSE	EHR	mimic_hosp.labevents
41	MAGNESIUM	EHR	mimic_hosp.labevents
42	CALCIUM, TOTAL	EHR	mimic_hosp.labevents
43	PHOSPHATE	EHR	mimic_hosp.labevents
44	INR(PT)	EHR	mimic_hosp.labevents
45	PT	EHR	mimic_hosp.labevents
46	PTT	EHR	mimic_hosp.labevents
47	LYMPHOCYTES	EHR	mimic_hosp.labevents
48	MONOCYTES	EHR	mimic_hosp.labevents
49	NEUTROPHILS	EHR	mimic_hosp.labevents
50	BASOPHILS	EHR	mimic_hosp.labevents
51	EOSINOPHILS	EHR	mimic_hosp.labevents
52	PH	EHR	mimic_hosp.labevents
53	BASE EXCESS	EHR	mimic_hosp.labevents
54	CALCULATED TOTAL CO2	EHR	mimic_hosp.labevents
55	PCO2	EHR	mimic_hosp.labevents
56	SPECIFIC GRAVITY	EHR	mimic_hosp.labevents
57	LACTATE	EHR	mimic_hosp.labevents
58	ALANINE AMINOTRANSFERASE (ALT)	EHR	mimic_hosp.labevents
59	ASPARATE AMINOTRANSFERASE (AST)	EHR	mimic_hosp.labevents
60	ALKALINE PHOSPHATASE	EHR	mimic_hosp.labevents
61	ALBUMIN	EHR	mimic_hosp.labevents
62	ArterialBloodPressurediastolic	EHR	mimic_icu.chartevents
63	ArterialBloodPressuremean	EHR	mimic_icu.chartevents
64	RespiratoryRate	EHR	mimic_icu.chartevents
65	AlarmsOn	EHR	mimic_icu.chartevents
66	MinuteVolumeAlarm-Low	EHR	mimic_icu.chartevents
67	Peakinsp.Pressure	EHR	mimic_icu.chartevents
68	PEEPset	EHR	mimic_icu.chartevents
69	MinuteVolume	EHR	mimic_icu.chartevents
70	TidalVolume(observed)	EHR	mimic_icu.chartevents
71	MinuteVolumeAlarm-High	EHR	mimic_icu.chartevents
72	MeanAirwayPressure	EHR	mimic_icu.chartevents
73	CentralVenousPressure	EHR	mimic_icu.chartevents
74	RespiratoryRate(Set)	EHR	mimic_icu.chartevents
75	PulmonaryArteryPressuremean	EHR	mimic_icu.chartevents
76	O2Flow	EHR	mimic_icu.chartevents
77	Glucosefingerstick	EHR	mimic_icu.chartevents
78	HeartRateAlarm-Low	EHR	mimic_icu.chartevents
79	PulmonaryArteryPressuresystolic	EHR	mimic_icu.chartevents
80	TidalVolume(set)	EHR	mimic_icu.chartevents
81	PulmonaryArteryPressurediastolic	EHR	mimic_icu.chartevents
82	SpO2DesatLimit	EHR	mimic_icu.chartevents
83	RespAlarm-High	EHR	mimic_icu.chartevents
84	SkinCare	EHR	mimic_icu.chartevents
85	Gcsverbal	EHR	mimic_icu.chartevents
86	Gcsmotor	EHR	mimic_icu.chartevents
87	Gcseyes	EHR	mimic_icu.chartevents
88	Systolic_blood_pressure_abp_mean	EHR	mimic_icu.chartevents
89	Heart_rate	EHR	mimic_icu.chartevents
90	Body_temperature	EHR	mimic_icu.chartevents
91	Pao2	EHR	mimic_hosp.labevents
92	Fio2	EHR	mimic_hosp.labevents
93	Urinary_output_sum	EHR	mimic_icu.outputevents
94	Serum_urea_nitrogen_level	EHR	mimic_hosp.labevents
95	White_blood_cells_count_mean	EHR	mimic_hosp.labevents
96	Serum_bicarbonate_level_mean	EHR	mimic_hosp.labevents
97	Sodium_level_mean	EHR	mimic_hosp.labevents
98	Potassium_level_mean	EHR	mimic_hosp.labevents
99	Bilirubin_level	EHR	mimic_hosp.labevents
100	ie_ratio_mean	EHR	mimic_icu.chartevents
101	Diastolic_blood_pressure_mean	EHR	mimic_icu.chartevents
102	Arterial_pressure_mean	EHR	mimic_icu.chartevents
103	Respiratory_rate	EHR	mimic_icu.chartevents
104	Spo2_peripheral	EHR	mimic_icu.chartevents
105	Glucose	EHR	mimic_icu.chartevents
106	Weight	EHR	mimic_icu.chartevents
107	Height	EHR	mimic_icu.chartevents
108	Hgb	EHR	mimic_hosp.labevents
109	Platelet	EHR	mimic_hosp.labevents
110	Chloride	EHR	mimic_hosp.labevents
111	Creatinine	EHR	mimic_hosp.labevents
112	Norepinephrine	EHR	mimic_icu.chartevents
113	Epinephrine	EHR	mimic_icu.chartevents
114	Phenylephrine	EHR	mimic_icu.chartevents
115	Vasopressin	EHR	mimic_icu.chartevents
116	Dopamine	EHR	mimic_icu.chartevents
117	Midazolam	EHR	mimic_icu.chartevents
118	Fentanyl	EHR	mimic_icu.chartevents
119	Propofol	EHR	mimic_icu.chartevents
120	Peep	EHR	mimic_hosp.labevents
121	Ph	EHR	mimic_hosp.labevents
122	Age	Demographic	mimic_core.patients, mimic_icu.icustays
123	AIDS	Comorbidity	mimic_hosp.diagnoses_icd
124	HEM	Comorbidity	mimic_hosp.diagnoses_icd
125	METS	Comorbidity	mimic_hosp.diagnoses_icd
126	AdmissionType_mimic3_processed	Admission	mimic_core.admissions
127	Gender	Demographic	mimic_core.patients
128	Admission_type	Admission	mimic_core.admissions
129	Admission_location	Admission	mimic_core.admissions
130	Insurance	Admission	mimic_core.admissions
131	Language	Demographic	mimic_core.admissions
132	Marital_status	Demographic	mimic_core.admissions
133	Ethnicity	Demographic	mimic_core.admissions
134	Congestive_heart_failure	Comorbidity	mimic_hosp.diagnoses_icd
135	Cardiac_arrhythmias	Comorbidity	mimic_hosp.diagnoses_icd
136	Valvular_disease	Comorbidity	mimic_hosp.diagnoses_icd
137	Pulmonary_circulation	Comorbidity	mimic_hosp.diagnoses_icd
138	Peripheral_vascular	Comorbidity	mimic_hosp.diagnoses_icd
139	Hypertension	Comorbidity	mimic_hosp.diagnoses_icd
140	Paralysis	Comorbidity	mimic_hosp.diagnoses_icd
141	Other_neurological	Comorbidity	mimic_hosp.diagnoses_icd
142	Chronic_pulmonary	Comorbidity	mimic_hosp.diagnoses_icd
143	Diabetes_uncomplicated	Comorbidity	mimic_hosp.diagnoses_icd
144	Diabetes_complicated	Comorbidity	mimic_hosp.diagnoses_icd
145	Hypothyroidism	Comorbidity	mimic_hosp.diagnoses_icd
146	Renal_failure	Comorbidity	mimic_hosp.diagnoses_icd
147	Liver_disease	Comorbidity	mimic_hosp.diagnoses_icd
148	Peptic_ulcer	Comorbidity	mimic_hosp.diagnoses_icd
149	Aids	Comorbidity	mimic_hosp.diagnoses_icd
150	Lymphoma	Comorbidity	mimic_hosp.diagnoses_icd
151	Metastatic_cancer	Comorbidity	mimic_hosp.diagnoses_icd
152	Solid_tumor	Comorbidity	mimic_hosp.diagnoses_icd
153	Rheumatoid_arthritis	Comorbidity	mimic_hosp.diagnoses_icd
154	Coagulopathy	Comorbidity	mimic_hosp.diagnoses_icd
155	Obesity	Comorbidity	mimic_hosp.diagnoses_icd
156	Weight_loss	Comorbidity	mimic_hosp.diagnoses_icd
157	Fluid_electrolyte	Comorbidity	mimic_hosp.diagnoses_icd
158	Blood_loss_anemia	Comorbidity	mimic_hosp.diagnoses_icd
159	Deficiency_anemias	Comorbidity	mimic_hosp.diagnoses_icd
160	Alcohol_abuse	Comorbidity	mimic_hosp.diagnoses_icd
161	Drug_abuse	Comorbidity	mimic_hosp.diagnoses_icd
162	Psychoses	Comorbidity	mimic_hosp.diagnoses_icd
163	Depression	Comorbidity	mimic_hosp.diagnoses_icd

### Distributions of demographic, admission, and comorbidity features

See Table 9.

**Table 9:** Distributions of demographic, admission and comorbidity features grouped by the in-hospital mortality label.

Feature	Value Name	In-hospital Mortality = 0	In-hospital Mortality = 1	All
Age	[.25, .50, .75] quantile	54.51, 66.63, 77.94	62.14, 74.44, 84.20	55.02, 67.13, 78.59
AIDS y	Negative	99.57% [39,644]	99.50% [3172]	99.56% [42,816]
	Positive	0.43% [173]	0.50% [16]	0.44% [189]
HEM	Negative	97.67% [38,890]	94.76% [3021]	97.46% [41,911]
	Positive	2.33% [927]	5.24% [167]	2.54% [1094]
METS	Negative	94.81% [37,749]	86.89% [2770]	94.22% [40,519]
	Positive	5.19% [2068]	13.11% [418]	5.78% [2486]
AdmissionType_mimic3_processed	medical	68.17% [27,145]	83.28% [2655]	69.29% [29,800]
	scheduled	3.57% [1420]	0.44% [14]	3.33% [1434]
	unscheduled	28.26% [11,252]	16.28% [519]	27.37% [11,771]
Gender	F	43.81% [17,443]	47.15% [1503]	44.06% [18,946]
	M	56.19% [22,374]	52.85% [1685]	55.94% [24,059]
Admission_type	AMBULATORY OBSERVATION	0.02% [7]	0.00% [0]	0.02% [7]
	DIRECT EMER.	3.56% [1418]	3.23% [103]	3.54% [1521]
	DIRECT OBSERVATION	0.09% [37]	0.06% [2]	0.09% [39]
	ELECTIVE	4.39% [1749]	0.78% [25]	4.13% [1774]
	EU OBSERVATION	0.22% [86]	0.03% [1]	0.20% [87]
	EW EMER.	50.37% [20,057]	61.48% [1960]	51.20% [22,017]
	OBSERVATION ADMIT	10.53% [4192]	10.04% [320]	10.49% [4512]
	SURGICAL SAME DAY ADMISSION	12.66% [5040]	1.35% [43]	11.82% [5083]
	URGENT	18.16% [7231]	23.02% [734]	18.52% [7965]
Admission_location	AMBULATORY SURGERY TRANSFER	0.06% [23]	0.03% [1]	0.06% [24]
	CLINIC REFERRAL	0.83% [330]	1.60% [51]	0.89% [381]
	EMERGENCY ROOM	48.93% [19,483]	58.91% [1878]	49.67% [21,361]
	INFORMATION NOT AVAILABLE	0.29% [117]	0.41% [13]	0.30% [130]
	INTERNAL TRANSFER TO OR FROM PSYCH	0.01% [3]	0.00% [0]	0.01% [3]
	PACU	0.56% [222]	0.31% [10]	0.54% [232]
	PHYSICIAN REFERRAL	24.22% [9643]	7.59% [242]	22.99% [9885]
	PROCEDURE SITE	1.69% [673]	0.53% [17]	1.60% [690]
	TRANSFER FROM HOSPITAL	21.20% [8442]	27.51% [877]	21.67% [9319]
	TRANSFER FROM SKILLED NURSING FACILITY	0.74% [293]	1.54% [49]	0.80% [342]
	WALK-IN/SELF REFERRAL	1.48% [588]	1.57% [50]	1.48% [638]
Insurance	Medicaid	7.15% [2846]	5.96% [190]	7.06% [3036]
	Medicare	42.77% [17,029]	54.80% [1747]	43.66% [18,776]
	Other	50.08% [19,942]	39.24% [1251]	49.28% [21,193]
Language	?	9.78% [3894]	10.92% [348]	9.86% [4242]
	ENGLISH	90.22% [35,923]	89.08% [2840]	90.14% [38,763]
Marital_status	DIVORCED	7.17% [2855]	5.58% [178]	7.05% [3033]
	MARRIED	46.68% [18,588]	40.15% [1280]	46.20% [19,868]
	None	6.63% [2641]	17.57% [560]	7.44% [3201]
	SINGLE	27.08% [10,784]	19.64% [626]	26.53% [11,410]
	WIDOWED	12.43% [4949]	17.06% [544]	12.77% [5493]
Ethnicity	AMERICAN INDIAN/ALASKA NATIVE	0.17% [66]	0.06% [2]	0.16% [68]
	ASIAN	2.88% [1147]	3.26% [104]	2.91% [1251]
	BLACK/AFRICAN AMERICAN	9.31% [3708]	7.34% [234]	9.17% [3942]
	HISPANIC/LATINO	3.60% [1432]	2.51% [80]	3.52% [1512]
	OTHER	4.81% [1914]	4.14% [132]	4.76% [2046]
	UNABLE TO OBTAIN	1.33% [531]	2.20% [70]	1.40% [601]
	UNKNOWN	9.06% [3609]	20.14% [642]	9.88% [4251]
	WHITE	68.84% [27,410]	60.35% [1924]	68.21% [29,334]
Congestive_heart_failure	Negative	98.66% [39,284]	98.90% [3153]	98.68% [42,437]
	Positive	1.34% [533]	1.10% [35]	1.32% [568]
Cardiac_arrhythmias	Negative	99.56% [39,640]	99.97% [3187]	99.59% [42,827]
	Positive	0.44% [177]	0.03% [1]	0.41% [178]
Valvular_disease	Negative	96.11% [38,267]	99.50% [3172]	96.36% [41,439]
	Positive	3.89% [1550]	0.50% [16]	3.64% [1566]
Pulmonary_circulation	Negative	99.37% [39,568]	99.34% [3167]	99.37% [42,735]
	Positive	0.63% [249]	0.66% [21]	0.63% [270]
Peripheral_vascular	Negative	98.78% [39,333]	98.78% [3149]	98.78% [42,482]
	Positive	1.22% [484]	1.22% [39]	1.22% [523]
Hypertension	Negative	99.68% [39,689]	99.91% [3185]	99.70% [42,874]
	Positive	0.32% [128]	0.09% [3]	0.30% [131]
Paralysis	Negative	100.00% [39,816]	99.97% [3187]	100.00% [43,003]
	Positive	0.00% [1]	0.03% [1]	0.00% [2]
Other_neurological	Negative	99.40% [39,577]	99.72% [3179]	99.42% [42,756]
	Positive	0.60% [240]	0.28% [9]	0.58% [249]
Chronic_pulmonary	Negative	99.52% [39,625]	99.78% [3181]	99.54% [42,806]
	Positive	0.48% [192]	0.22% [7]	0.46% [199]
Diabetes_uncomplicated	Negative	99.40% [39,578]	99.97% [3187]	99.44% [42,765]
	Positive	0.60% [239]	0.03% [1]	0.56% [240]
Diabetes_complicated	Negative	99.73% [39,710]	99.87% [3184]	99.74% [42,894]
	Positive	0.27% [107]	0.13% [4]	0.26% [111]
Hypothyroidism	Negative	99.99% [39,813]	100.00% [3188]	99.99% [43,001]
	Positive	0.01% [4]	0.00% [0]	0.01% [4]
Renal_failure	Negative	99.88% [39,768]	99.97% [3187]	99.88% [42,955]
	Positive	0.12% [49]	0.03% [1]	0.12% [50]
Liver_disease	Negative	99.45% [39,597]	99.25% [3164]	99.43% [42,761]
	Positive	0.55% [220]	0.75% [24]	0.57% [244]
Peptic_ulcer	Negative	99.97% [39,805]	100.00% [3188]	99.97% [42,993]
	Positive	0.03% [12]	0.00% [0]	0.03% [12]
Aids	Negative	99.90% [39,776]	99.84% [3183]	99.89% [42,959]
	Positive	0.10% [41]	0.16% [5]	0.11% [46]
Lymphoma	Negative	99.80% [39,739]	99.40% [3169]	99.77% [42,908]
	Positive	0.20% [78]	0.60% [19]	0.23% [97]
Metastatic_cancer	Negative	99.09% [39,456]	98.84% [3151]	99.07% [42,607]
	Positive	0.91% [361]	1.16% [37]	0.93% [398]
Solid_tumor	Negative	97.80% [38,943]	97.24% [3100]	97.76% [42,043]
	Positive	2.20% [874]	2.76% [88]	2.24% [962]
Rheumatoid_arthritis	Negative	99.97% [39,805]	99.87% [3184]	99.96% [42,989]
	Positive	0.03% [12]	0.13% [4]	0.04% [16]
Coagulopathy	Negative	99.96% [39,801]	99.97% [3187]	99.96% [42,988]
	Positive	0.04% [16]	0.03% [1]	0.04% [17]
Obesity	Negative	99.94% [39,792]	100.00% [3188]	99.94% [42,980]
	Positive	0.06% [25]	0.00% [0]	0.06% [25]
Weight_loss	Negative	99.99% [39,813]	100.00% [3188]	99.99% [43,001]
	Positive	0.01% [4]	0.00% [0]	0.01% [4]
Fluid_electrolyte	Negative	99.66% [39,682]	99.87% [3184]	99.68% [42,866]
	Positive	0.34% [135]	0.13% [4]	0.32% [139]
Blood_loss_anemia	Negative	99.97% [39,807]	99.97% [3187]	99.97% [42,994]
	Positive	0.03% [10]	0.03% [1]	0.03% [11]
Deficiency_anemias	Negative	99.98% [39,808]	100.00% [3188]	99.98% [42,996]
	Positive	0.02% [9]	0.00% [0]	0.02% [9]
Alcohol_abuse	Negative	99.54% [39,635]	100.00% [3188]	99.58% [42,823]
	Positive	0.46% [182]	0.00% [0]	0.42% [182]
Drug_abuse	Negative	99.94% [39,794]	100.00% [3188]	99.95% [42,982]
	Positive	0.06% [23]	0.00% [0]	0.05% [23]
Psychoses	Negative	99.97% [39,807]	100.00% [3188]	99.98% [42,995]
	Positive	0.03% [10]	0.00% [0]	0.02% [10]
Depression	Negative	100.00% [39,817]	100.00% [3188]	100.00% [43,005]
	Positive	0.00% [0]	0.00% [0]	0.00% [0]

### Model Performance w.r.t Feature Drop Ratio

See Fig. 8.

### Visualization of Global Feature Importance Ranks

See Figs. 9, 10, 11, 12, 13 and 14.

### Figure for Interpretability and Fairness Interactions

see Fig. 15

### Feature Interpretability for Indicating Unfairness

We use a simple and synthetic medical dataset to illustrate how model interpretability can lead to correcting unfairness. In our dataset, we have a feature X which we use as an indicator of sickness. We have a sensitive feature S representing the groups we are trying not to discriminate over. We have a treatment T which will help those who are actually sick have a much lower chance of mortality ($10\%$) rather than the expected ($50\%$) if the disease is left untreated. We assume that the disease is monotonically more likely with the symptom X ($\sigma (5x-5)$). First, we only treat those patients who are very sick, with X greater than one. Second, we condition the dataset to be specifically unfair, treating only 40% of those with S = 1 and 80% of those with S = 0, see Figure 16. Despite the relatively minimal effect this has on the overall mortality by sensitive group, the model has learned to pick up directly on this important characteristic. We train a model both for a dataset (*X*, *S*) where the model has no idea about the latent treatment *T* and also for a dataset which includes (*X*, *S*, *T*). In the first case, the model must explicitly depend on the discrimination against the group $S=1$ to achieve optimal predictions, whereas in the second dataset, the model is able to condition on the treatment T instead of the sensitive feature S.

For our experiments we generated 70,000 samples and trained on 80% of the data. For our model, we trained a deep neural network with hidden layers of sizes (256,128,64) with input layers of size 2 and 3 and output layers of size 1. We used a sigmoid activation and mean-squared error loss with a learning rate of 5e-3.

In the above Fig. 17, we can see on the left how when T is hidden from the model, it explicitly depends on the biases inherited from the latent variable for its predictions. We can also see how this is drastically reduced on the right when we include the latent variable which is the cause of the bias. Regardless, we still see that the blue curves remain above the red curves both for the treated and untreated populations. The model still tries to picks up on the correlations of greater deaths in the S=1 population and hence has a slight upwards bias for this group.

These experiments portray a clear case where interpretability helps point out the unfairness of a model. First spotting the feature importance of the sensitive attribute S would indicate to a practitioner that there is some type of different treatment by group of S. Next, seeing the curve on the left would allow a practitioner to realize that there is some larger bias within the dataset that they are training with. Finally, adding the treatment T to the model and realizing the underlying T variable is holding all of the bias from the underlying data generation process allows them to not only spot the cause of the bias, but mostly eliminate the bias from their prediction model.

## References

[1] Purushotham S, Meng C, Che Z, Liu Y (2018). Benchmarking deep learning models on large healthcare datasets. J. Biomed. Inf..

[2] Harutyunyan H, Khachatrian H, Kale DC, Ver Steeg G, Galstyan A (2019). Multitask learning and benchmarking with clinical time series data. Sci. Data.

[3] Wang, S. *et al.* Mimic-extract: A data extraction, preprocessing, and representation pipeline for mimic-iii. In *Proceedings of the ACM Conference on Health, Inference, and Learning*, 222–235 (2020).

[4] Chen, I., Johansson, F. D. & Sontag, D. Why is my classifier discriminatory? In *Adv. Neural Inf. Process. Syst.*, 3539–3550 (2018).

[5] Johnson, A. *et al.* Mimic-iv (version 0.4). *PhysioNet* (2020).

[6] Goldberger A (2000). Physiobank, physiotoolkit, and physionet: Components of a new research resource for complex physiologic signals. Circulation [Online].

[7] Hooker, S., Erhan, D., Kindermans, P.-J. & Kim, B. A benchmark for interpretability methods in deep neural networks. *Adv. Neural Inf. Process. Syst.*, 9737–9748 (2019).

[8] Tsang M, Rambhatla S, Liu Y (2020). How does this interaction affect me? Interpretable attribution for feature interactions. Adv. Neural Inf. Process. Syst..

[9] Simonyan, K., Vedaldi, A. & Zisserman, A. Deep inside convolutional networks: Visualising image classification models and saliency maps. arXiv preprint arXiv:1312.6034 (2013).

[10] Sundararajan, M., Taly, A. & Yan, Q. Axiomatic attribution for deep networks. In *Proceedings of the 34th International Conference on Machine Learning-Volume 70*, 3319–3328 (2017).

[11] Shrikumar, A., Greenside, P. & Kundaje, A. Learning important features through propagating activation differences. In *International Conference on Machine Learning*, 3145–3153 (2017).

[12] Ancona, M., Ceolini, E., Öztireli, C. & Gross, M. Towards better understanding of gradient-based attribution methods for deep neural networks. In *International Conference on Learning Representations* (2018).

[13] Lundberg, S. M. & Lee, S.-I. A unified approach to interpreting model predictions. *Adv. Neural Inf. Process. Syst.*, 4765–4774 (2017).

[14] Smilkov, D., Thorat, N., Kim, B., Viégas, F. & Wattenberg, M. Smoothgrad: removing noise by adding noise. arXiv preprint arXiv:1706.03825 (2017).

[15] Castro J, Gómez D, Tejada J (2009). Polynomial calculation of the shapley value based on sampling. Comput. Oper. Res..

[16] Strumbelj E, Kononenko I (2010). An efficient explanation of individual classifications using game theory. J. Mach. Learn. Res..

[17] Molnar C (2020). Interpretable Machine Learning.

[18] Suresh, H. *et al.* Clinical intervention prediction and understanding using deep networks. arXiv preprint arXiv:1705.08498 (2017).

[19] Zeiler, M. D. & Fergus, R. Visualizing and Understanding Convolutional Networks. In *European conference on computer vision*, 818–833 (Springer, 2014).

[20] Sundararajan, M., Dhamdhere, K. & Agarwal, A. The shapley taylor interaction index. In *International Conference on Machine Learning*, 9259–9268 (PMLR, 2020).

[21] Janizek, J. D., Sturmfels, P. & Lee, S.-I. Explaining explanations: Axiomatic feature interactions for deep networks. arXiv preprint arXiv:2002.04138 (2020).

[22] Sorokina, D., Caruana, R., Riedewald, M. & Fink, D. Detecting statistical interactions with additive groves of trees. In *Proceedings of the 25th international conference on Machine learning*, 1000–1007 (2008).

[23] Tsang, M., Cheng, D. & Liu, Y. Detecting statistical interactions from neural network weights. In *International Conference on Learning Representations* (2018).

[24] Tsang, M., Liu, H., Purushotham, S., Murali, P. & Liu, Y. Neural interaction transparency (nit): Disentangling learned interactions for improved interpretability. *Adv. Neural Inf. Process. Syst.*, 5804–5813 (2018).

[25] Dhamdhere, K., Sundararajan, M. & Yan, Q. How important is a neuron? arXiv preprint arXiv:1805.12233 (2018).

[26] Shrikumar, A., Su, J. & Kundaje, A. Computationally efficient measures of internal neuron importance. arXiv preprint arXiv:1807.09946 (2018).

[27] Leino, K., Sen, S., Datta, A., Fredrikson, M. & Li, L. Influence-directed explanations for deep convolutional networks. In *2018 IEEE International Test Conference (ITC)*, 1–8 (IEEE, 2018).

[28] Springenberg, J., Dosovitskiy, A., Brox, T. & Riedmiller, M. Striving for simplicity: The all convolutional net. In *ICLR (workshop track)* (2015).

[29] Kim, B. *et al.* Interpretability beyond feature attribution: Quantitative testing with concept activation vectors (tcav). In *International Conference on Machine Learning*, 2668–2677 (PMLR, 2018).

[30] Ghorbani, A., Wexler, J., Zou, J. Y. & Kim, B. Towards automatic concept-based explanations. *Adv. Neural Inf. Process. Syst.*, 9277–9286 (2019).

[31] Zhou, B., Sun, Y., Bau, D. & Torralba, A. Interpretable basis decomposition for visual explanation. In *Proceedings of the European Conference on Computer Vision (ECCV)*, 119–134 (2018).

[32] Ismail AA, Gunady M, Corrada Bravo H, Feizi S (2020). Benchmarking deep learning interpretability in time series predictions. Adv. Neural Inf. Process. Syst..

[33] Hardt, M. *et al.* Explaining an increase in predicted risk for clinical alerts. In *Proceedings of the ACM Conference on Health, Inference, and Learning*, 80–89 (2020).

[34] Sanchez-Lengeling B (2020). Evaluating attribution for graph neural networks. Adv. Neural Inf. Process. Syst..

[35] Samek W, Binder A, Montavon G, Lapuschkin S, Müller K-R (2016). Evaluating the visualization of what a deep neural network has learned. IEEE Trans. Neural Netw. Learn. Syst..

[36] Lambrecht A, Tucker C (2019). Algorithmic bias? an empirical study of apparent gender-based discrimination in the display of stem career ads. Manage. Sci..

[37] Raji, I. D. & Buolamwini, J. Actionable auditing: Investigating the impact of publicly naming biased performance results of commercial ai products. In *Proceedings of the 2019 AAAI/ACM Conference on AI, Ethics, and Society*, 429–435 (2019).

[38] Schnabel, T., Swaminathan, A., Singh, A., Chandak, N. & Joachims, T. Recommendations as treatments: Debiasing learning and evaluation. arXiv preprint arXiv:1602.05352 (2016).

[39] Dressel J, Farid H (2018). The accuracy, fairness, and limits of predicting recidivism. Sci. Adv..

[40] Fu, R., Huang, Y. & Singh, P. V. Artificial intelligence and algorithmic bias: Source, detection, mitigation, and implications. In *Pushing the Boundaries: Frontiers in Impactful OR/OM Research*, 39–63 (INFORMS, 2020).

[41] Mehrabi, N., Morstatter, F., Saxena, N., Lerman, K. & Galstyan, A. A survey on bias and fairness in machine learning. arXiv preprint arXiv:1908.09635 (2019).

[42] Hardt, M., Price, E. & Srebro, N. Equality of opportunity in supervised learning. *Adv. Neural Inf. Process. Syst.*, 3315–3323 (2016).

[43] Bellamy, R. K. *et al.* Ai fairness 360: An extensible toolkit for detecting, understanding, and mitigating unwanted algorithmic bias. arXiv preprint arXiv:1810.01943 (2018).

[44] Kamiran F, Calders T (2012). Data preprocessing techniques for classification without discrimination. Knowl. Inf. Syst..

[45] Moyer D, Gao S, Brekelmans R, Galstyan A, Ver Steeg G (2018). Invariant representations without adversarial training. Adv. Neural Inf. Process. Syst..

[46] Singh, H., Singh, R., Mhasawade, V. & Chunara, R. Fair predictors under distribution shift. arXiv preprint arXiv:1911.00677 (2019).

[47] Barda N (2020). Addressing bias in prediction models by improving subpopulation calibration. J. Am. Med. Inf. Assoc..

[48] Martinez, N., Bertran, M. & Sapiro, G. Minimax pareto fairness: A multi objective perspective. In *International Conference on Machine Learning*, 6755–6764 (PMLR, 2020). PMC7912461 33644764

[49] Zhang, H., Lu, A. X., Abdalla, M., McDermott, M. & Ghassemi, M. Hurtful words: quantifying biases in clinical contextual word embeddings. In *Proceedings of the ACM Conference on Health, Inference, and Learning*, 110–120 (2020).

[50] Chen IY, Szolovits P, Ghassemi M (2019). Can AI help reduce disparities in general medical and mental health care?. AMA J. Ethics.

[51] Cui, S., Pan, W., Zhang, C. & Wang, F. xorder: A model agnostic post-processing framework for achieving ranking fairness while maintaining algorithm utility. arXiv preprint arXiv:2006.08267 (2020).

[52] Chen, J., Berlot-Atwell, I., Hossain, S., Wang, X. & Rudzicz, F. Exploring text specific and blackbox fairness algorithms in multimodal clinical nlp. arXiv preprint arXiv:2011.09625 (2020).

[53] Sharma, S., Henderson, J. & Ghosh, J. Certifai: Counterfactual explanations for robustness, transparency, interpretability, and fairness of artificial intelligence models. arXiv preprint arXiv:1905.07857 (2019).

[54] Chu, E., Gillani, N. & Priscilla Makini, S. Games for fairness and interpretability. In *Companion Proceedings of the Web Conference 2020*, 520–524 (2020).

[55] Doshi-Velez, F. & Kim, B. A roadmap for a rigorous science of interpretability. arXiv preprint arXiv:1702.08608 **2** (2017).

[56] Lipton ZC (2018). The mythos of model interpretability. Queue.

[57] Du M, Yang F, Zou N, Hu X (2020). Fairness in deep learning: A computational perspective. IEEE Intell. Syst..

[58] Adebayo, J. & Kagal, L. Iterative orthogonal feature projection for diagnosing bias in black-box models. arXiv preprint arXiv:1611.04967 (2016).

[59] Wadsworth, C., Vera, F. & Piech, C. Achieving fairness through adversarial learning: an application to recidivism prediction. arXiv preprint arXiv:1807.00199 (2018).

[60] Cesaro, J. & Cozman, F. G. Measuring unfairness through game-theoretic interpretability. In *Joint European Conference on Machine Learning and Knowledge Discovery in Databases*, 253–264 (Springer, 2019).

[61] Kleinberg, J. & Mullainathan, S. Simplicity creates inequity: implications for fairness, stereotypes, and interpretability. In *Proceedings of the 2019 ACM Conference on Economics and Computation*, 807–808 (2019).

[62] Jabbari, S., Ou, H.-C., Lakkaraju, H. & Tambe, M. An empirical study of the trade-offs between interpretability and fairness. *ICML 2020 Workshop on Human Interpretability in Machine Learning* (2020).

[63] Wang, C., Han, B., Patel, B., Mohideen, F. & Rudin, C. In pursuit of interpretable, fair and accurate machine learning for criminal recidivism prediction. arXiv preprint arXiv:2005.04176 (2020).

[64] Sjoding, M. *et al.* Democratizing ehr analyses a comprehensive pipeline for learning from clinical data. *Machine Learning For Healthcare (Clinical Abstracts Track)* (2019).

[65] Song, W. *et al.* Autoint: Automatic feature interaction learning via self-attentive neural networks. In *Proceedings of the 28th ACM International Conference on Information and Knowledge Management*, 1161–1170 (2019).

[66] Hochreiter S, Schmidhuber J (1997). Long short-term memory. Neural Comput..

[67] Bai, S., Kolter, J. Z. & Koltun, V. An empirical evaluation of generic convolutional and recurrent networks for sequence modeling. arXiv preprint arXiv:1803.01271 (2018).

[68] Vaswani A (2017). Attention is all you need. Adv. Neural Inf. Process. Syst..

[69] Guo, T., Lin, T. & Antulov-Fantulin, N. Exploring interpretable lstm neural networks over multi-variable data. In *International Conference on Machine Learning*, 2494–2504 (2019).

[70] Jain, S. & Wallace, B. C. Attention is not explanation. In *Proceedings of the 2019 Conference of the North American Chapter of the Association for Computational Linguistics: Human Language Technologies, Volume 1 (Long and Short Papers)*, 3543–3556 (2019).

[71] Grimsley, C., Mayfield, E. & R.S. Bursten, J. Why attention is not explanation: Surgical intervention and causal reasoning about neural models. In *Proceedings of the 12th Language Resources and Evaluation Conference*, 1780–1790 (European Language Resources Association, Marseille, France, 2020).

[72] Knaus WA (1991). The apache iii prognostic system: Risk prediction of hospital mortality for critically iii hospitalized adults. Chest.

[73] Le Gall J-R (1996). The logistic organ dysfunction system: A new way to assess organ dysfunction in the intensive care unit. Jama.

[74] Johnson AE, Kramer AA, Clifford GD (2013). A new severity of illness scale using a subset of acute physiology and chronic health evaluation data elements shows comparable predictive accuracy. Crit. Care Med..

[75] Le Gall J-R, Lemeshow S, Saulnier F (1993). A new simplified acute physiology score (saps ii) based on a European/north American multicenter study. Jama.

[76] Bone RC (1992). Definitions for sepsis and organ failure and guidelines for the use of innovative therapies in sepsis. Chest.

[77] Vincent, J.-L. *et al.* The sofa (sepsis-related organ failure assessment) score to describe organ dysfunction/failure (1996). 10.1007/BF01709751 8844239

[78] Wong, A., Wang, X. Y. & Hryniowski, A. How much can we really trust you? towards simple, interpretable trust quantification metrics for deep neural networks. arXiv preprint arXiv:2009.05835 (2020).

[79] Cheng M, Nazarian S, Bogdan P (2020). There is hope after all: Quantifying opinion and trustworthiness in neural networks. Front. Artif. Intell..

[80] Chen, J., Kallus, N., Mao, X., Svacha, G. & Udell, M. Fairness under unawareness: Assessing disparity when protected class is unobserved. In *Proceedings of the conference on fairness, accountability, and transparency*, 339–348 (2019).

[81] Yarnell CJ (2017). Association between immigrant status and end-of-life care in Otario, Canada. JAMA.

[82] Lee JJ, Long AC, Curtis JR, Engelberg RA (2016). The influence of race/ethnicity and education on family ratings of the quality of dying in the ICU. J. Pain Symp. Manage..

[83] Nelson A (2002). Unequal treatment: Confronting racial and ethnic disparities in health care. J. Natl. Med. Assoc..

[84] Rubin MA, Dhar R, Diringer MN (2014). Racial differences in withdrawal of mechanical ventilation do not alter mortality in neurologically injured patients. J. Crit. Care.

[85] Kleinberg, J., Mullainathan, S. & Raghavan, M. Inherent trade-offs in the fair determination of risk scores. arXiv preprint arXiv:1609.05807 (2016).

[86] Lahoti P (2021). Fairness without demographics through adversarially reweighted learning. Adv. Neural Inf. Process. Syst..

[87] Corbett-Davies, S. & Goel, S. The measure and mismeasure of fairness: A critical review of fair machine learning. arXiv preprint arXiv:1808.00023 (2018).

[88] Hunter JD (2007). Matplotlib: A 2d graphics environment. Computi. Sci. Eng..

